# Did I do that? Detecting a perturbation to visual feedback in a reaching task

**DOI:** 10.1167/19.1.5

**Published:** 2019-01-14

**Authors:** Elon Gaffin-Cahn, Todd E Hudson, Michael S Landy

**Affiliations:** eg.gc@nyu.edu; Todd.Hudson@nyumc.org; http://www.cns.nyu.edu/~msl landy@nyu.edu; Department of Psychology, New York University, New York, NY, USA; Departments of Neurology and Rehabilitation Medicine, New York University Langone Medical Center, New York, NY, USA; Departments of Psychology and Center for Neural Science, New York University, New York, NY, USA

**Keywords:** *sensorimotor*, *error detection*, *cue combination*, *ideal observer*, *feedback perturbation*

## Abstract

The motor system executes actions in a highly stereotyped manner despite the high number of degrees of freedom available. Studies of motor adaptation leverage this fact by disrupting, or perturbing, visual feedback to measure how the motor system compensates. To elicit detectable effects, perturbations are often large compared to trial-to-trial reach endpoint variability. However, awareness of large perturbations can elicit qualitatively different compensation processes than unnoticeable ones can. The current experiment measures the perturbation detection threshold, and investigates how humans combine proprioception and vision to decide whether displayed reach endpoint errors are self-generated only, or are due to experimenter-imposed perturbation. We scaled or rotated the position of the visual feedback of center-out reaches to targets and asked subjects to indicate whether visual feedback was perturbed. Subjects detected perturbations when they were at least 1.5 times the standard deviation of trial-to-trial endpoint variability. In contrast to previous studies, subjects suboptimally combined vision and proprioception. Instead of using proprioceptive input, they responded based on the final (possibly perturbed) visual feedback. These results inform methodology in motor system experimentation, and more broadly highlight the ability to attribute errors to one's own motor output and combine visual and proprioceptive feedback to make decisions.

## Introduction

The motor system is the medium through which we interact with the world. It is remarkably accurate and self-correcting despite the large set of factors that affect a motor plan in the pursuit of a specific goal. These factors can be external, such as the weight of a heavy tool, correcting for wind in a field goal attempt, or the introduction of an uneven reward landscape such as when reaching around a fragile wine glass. Motor plans can also be altered due to internal factors, such as fatigue or becoming more precise through learning. Through a lifetime of practice, people use visual and proprioceptive feedback to learn how to achieve a motor goal by updating motor commands in response to both external and internal constraints. We investigate how people combine these sensory cues to make judgments about the outcome of these motor commands.

Studies of movement planning typically examine the effects of internal and external factors by artificially manipulating the movement. Often, the manipulation interferes with the subject's naturally learned behavior. For example, to measure how a subject changes behavior to improve performance, a reach may be displaced mechanically (Hwang, Smith, & Shadmehr, 2006; Sanes & Evarts, 1983) or a visual indicator of the unseen hand is displaced (Held & Freedman, 1963; Hudson & Landy, 2012a, 2016; Mazzoni & Krakauer, 2006). These are effective ways of learning about the properties of the motor system because of the ease of implementation and the ability to measure compensation under various contexts and conditions.

These displacements of the reach or reach feedback, called perturbations, are often large and potentially noticeable. Noticing a perturbation of the reach endpoint may allow subjects to compensate using a conscious, top-down approach, which may be subject to cognitive biases (Harris, 1974) and is more difficult to accomplish under cognitive load (Ingram et al., 2000). Hwang et al. (2006) describe explicit and implicit motor compensation processes (i.e., compensation when aware or unaware of the manipulation) that work in parallel and both contribute to maintaining performance during perturbed movements. These processes operate with different learning rates, have different amounts of savings during subsequent re-adaptation, and can be weighted asymmetrically in subsequentnt motor plans (Huberdeau, Krakauer, & Haith, 2015; Taylor, Krakauer, & Ivry, 2014). Typical studies show less generalization and transfer of learning to the contralateral limb during adaptation than during an explicit change of strategy (e.g., Malfait & Ostry, 2004; but see also Torres-Oviedo & Bastian, 2012). Furthermore, these systems can operate in opposition to one another (Mazzoni & Krakauer, 2006) and are subserved by different neural circuitry (Galea, Vazquez, Pasricha, de Xivry, & Celnik, 2011; Taylor, Klemfuss, & Ivry, 2010). This suggests that motor adaptation is a qualitatively different process than consciously changing a motor goal. These processes directly inform real-world applications, including rehabilitation and skill learning, underscoring the need to learn more about how people detect perturbations.

Some studies attempt to avoid subjects noticing perturbations by increasing the perturbation magnitude over many smaller steps that build up to a large final value (Hudson, Lackner, & DiZio, 2005; Kagerer, Contreras-Vidal, & Stelmach, 1997; Kluzik, Diedrichsen, Shadmehr, & Bastian, 2008; Malfait & Ostry, 2004; Sawers, Kelly, & Hahn, 2013; Werner et al., 2015; Wong & Shelhamer, 2011), or by using a perturbation magnitude that fluctuates over trials (Cassanello, Ohl, & Rolfs, 2016; Hudson & Landy, 2012a). However, none have directly addressed the issue of perturbation detection. Werner et al. (2015) attempted to indirectly estimate detection ability by calculating an awareness index with the Process Dissociation Procedure (Jacoby, 1991). This allowed Werner et al. to estimate the degree to which reaches were driven by adaptation versus explicit strategies, but does not directly address the question of whether or not participants were able to detect the perturbation.

Detecting a perturbation is a consequence of the combination of sensory signals that provide information about the location of the end effector. The specific manner in which these signals are combined is an area of active research. Previous studies have shown that vision and proprioception are combined optimally in motor planning (Sober & Sabes, 2003, 2005; van Beers, Sittig, & van Der Gon, 1999; van Beers, Wolpert, & Haggard, 2002; van Dam & Ernst, 2013). This suggests that people may combine the outcome of a forward model of the reach, proprioception at reach endpoint, and the (possibly perturbed) visual feedback to make a statistically optimal inference. Similar results have been found in the oculomotor system (Collins, Rolfs, Deubel, & Cavanagh, 2009; Niemeier, Crawford, & Tweed, 2003; but see also Cavanaugh, Berman, Joiner, & Wurtz, 2016). Kluzik et al. (2008) found that inducing a gradual adaptation compels subjects to update their internal model of the arm rather than their model of an external tool, suggesting that the central nervous system (CNS) attributes smaller errors to itself. Berniker and Körding (2008) theorized that sensorimotor adaptation and certain instances of generalization in motor learning could be explained by a model of the CNS that selectively attributes motor error to either errors in self-generated torque or external perturbation (e.g., from a force field perturbation). However, empirical tests by Hudson and Landy (2012b) found that human subjects were unable to compensate for self-generated torques in a reflexively perturbed reaching task. None of these studies have investigated sensory integration for the purpose of detecting a perturbation.

**Figure 1:**
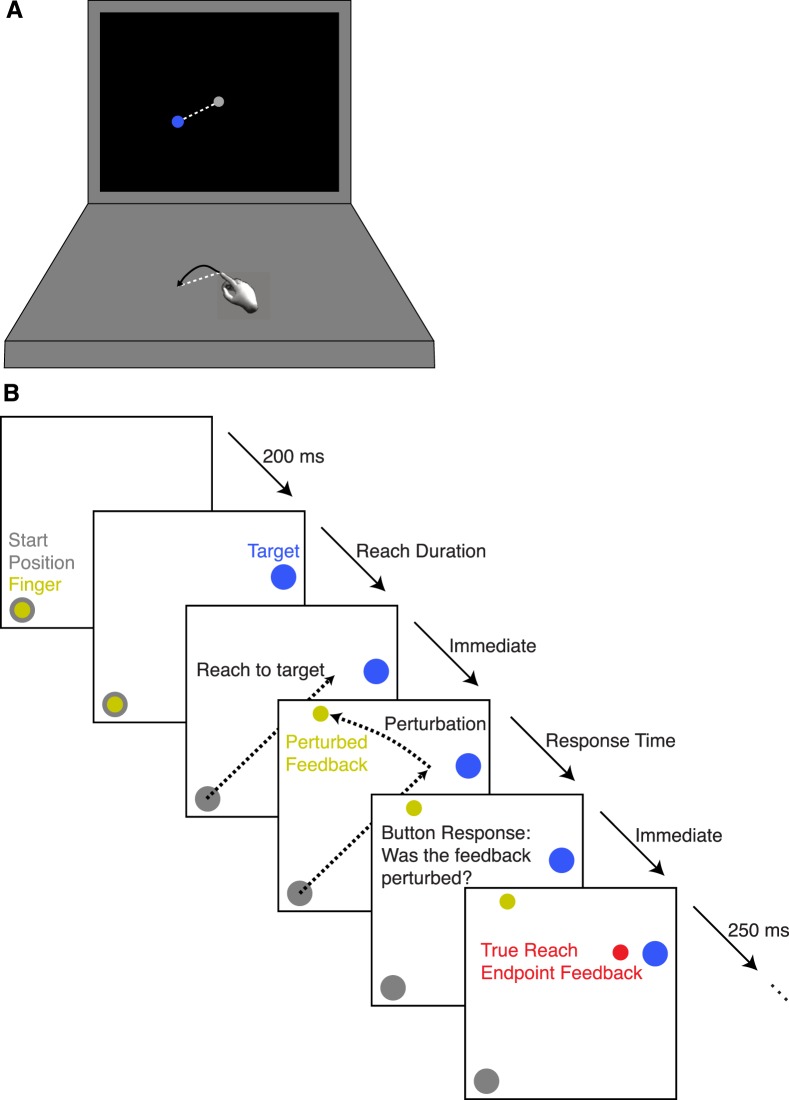
Experimental setup. (A) Diagram of the table and monitor. Subjects' fingers were tracked as they lifted and reached from one location to another on a horizontal tabletop (objective movement indicated by dotted white line). Visual display of the starting point (gray circle), target (blue circle), and reach endpoint was on a fronto-parallel display scaled identically to tabletop locations. Vertical hand movements were not represented on the screen. (B) Trial sequence. There was a 250 ms intertrial interval and a 200 ms delay before the target appeared. All other durations were controlled by the subject.

In the current experiment, we tested subjects' ability to detect perturbations applied to reach endpoints. Subjects made reaches across a horizontal table while task information and reach feedback was provided on a vertical fronto-parallel monitor. Small circles representing the target and fingertip were displayed on the screen (Figure 1A). On some trials, visual feedback of reach endpoint location was perturbed. Subjects indicated whether feedback was veridical or perturbed. This task allowed us to measure the ability to detect perturbation, both in absolute terms as well as relative to the participant's own motor variability. It also allowed us to investigate how people combine sensory signals. For this, we developed three models of behavior. The first model optimally combines knowledge of the distributions of noisy proprioceptive and visual signals. A second, suboptimal model combines point estimates of each source of information. The third model uses visual information exclusively. Previous literature suggests that proprioceptive and visual cues are combined optimally. Our data are inconsistent with this prediction: Participants only responded that they were perturbed when the visual feedback indicated that the reach error was large.

## Methods

### Participants

Five right-handed subjects (three female, two male; mean age 25.6 years) completed the experiment. Subjects were naive to the experimental goals, except for Subject 1, author EGC. All subjects were paid $10 per 45-min session and provided written consent in accordance with the New York University Institutional Review Board and the Declaration of Helsinki. Two additional subjects completed an initial practice session but did not continue because one failed to properly implement the task instructions, and one sustained an injury to the dominant hand in an accident unrelated to the research program.

### Apparatus

Subjects performed the task in a dimly lit room, seated against a table that extended 35.3 cm from a 21″ CRT monitor. Subjects wore a ring on the most distal knuckle of the right index finger with three infrared LED markers on both sides of the ring as part of an Optotrak 3020 dual-camera setup (Northern Digital Inc., Waterloo, Canada) to continuously track fingertip movements at 200 Hz. The horizontal plane was represented on the monitor such that viewing the monitor while seated was equivalent to viewing the table from above (Figure 1A). A calibration run at the beginning of each session allowed us to estimate the position of the fingertip based on the Optotrak markers. Start points, targets, and the fingertip position were represented on the display screen. Subjects placed the right index finger on the tabletop and made pointing movements to other locations on the table, as instructed by on-screen targets. Stimulus presentation was performed using PsychToolbox (Brainard, 1997; Pelli, 1997) and analyses were carried out using MATLAB (MathWorks, Natick, MA).

### Design

Subjects made reaching motions toward an eccentric target. Subjects completed 4000 total reaches, split over the eight sessions of 500 trials each (Subjects 2 and 5 completed one fewer trial due to premature termination of the program). Subjects who were unfamiliar with motor tasks in the lab performed an introductory 500-trial practice session to become acquainted with the setup and so that their average motor error would stabilize. All sessions began with an additional 200-trial warmup period. Trials from the introductory practice session and warmup periods had no perturbation and were only used for the purpose of yoking the perturbation magnitudes to each subject's individual motor variability. Each subject's motor variability was calculated as the standard deviation (*SD*) in gain or direction of finger endpoint error during the 200 practice trials at the beginning of the first session. In the main experimental portion of each session, endpoint feedback was perturbed on half the trials. Perturbation magnitudes were 1, 1.5, 2.2, 3.3, or 5 times the participant's motor *SD*. Perturbations were applied in both directions (stretching and shrinking in gain and clockwise and counterclockwise in direction) so that there were 10 possible perturbations per session. The 10 perturbations, along with the no-perturbation trials, were presented in random order to minimize corrections (i.e., adaptation). In the first four sessions reach gain was perturbed, and in the next four, reach direction was perturbed.

### Task

An example trial sequence is shown in Figure 1B. Subjects initiated each trial by moving the dominant (right) index finger until the finger indicator dot covered the central start circle. After a 200 ms delay, an auditory cue indicated the appearance of the target, a circle (0.4 cm diameter) that appeared 10.1 cm away from the start circle. The target remained on the screen until the trial was completed. Target direction was random and uniform across trials. Reach onset and offset were signaled with an auditory cue. Subjects were instructed to lift the finger off the table during the reach to avoid inhomogeneities in the friction of the tabletop, completing the reach by landing again on the table. This requirement made accurately assessing reach endpoint location easier. Movement onset was defined as the moment the finger indicator left the start circle and the vertical position of the finger elevated 0.2 mm. The location of the finger was not revealed on the screen during the trajectory to minimize effects of online feedback. Reach offset thresholds were developed from analysis of trajectory kinematics measured in pilot studies and from past datasets. Reach offset was defined as the moment when all of the following criteria were satisfied: (a) The fingertip was vertically lower than 2 mm above the starting position (note that the starting position was often based on the finger location after the finger had lifted up off the table), (b) the instantaneous vertical velocity had slowed sufficiently (less than 0.1 mm/s downward) or the finger began to rebound upward, (c) instantaneous vertical acceleration was greater than 0.01 mm/s^2^ upwards (i.e., the finger was decelerating downward), and (d) the reach was greater than two-thirds of the distance to the target to prevent egregious reaching errors. Trajectories were limited to 500 ms; otherwise the trial would end abruptly, and the subject would be notified of the timeout by text on the screen and an unpleasant beep. Trials were also discarded if the reach duration was less than 100 ms or if the finger data were missing for more than 50 ms, which indicated a threshold or tracking issue. Trials with any of these errors were repeated immediately with the same target.

Once the trajectory concluded, the feedback dot, either perturbed or veridical, appeared on the screen. Subjects then had unlimited time to indicate via key press using the nondominant (left) hand whether they believed the feedback had been perturbed. After the response, subjects received full feedback about the response and reach endpoint (Figure 1B). The screen then went blank for a 250 ms intertrial interval before the start circle reappeared. On practice and warmup trials, the reach endpoint was shown immediately after the reach, but there were no perturbations and no detection task. The experiment was self-paced. Each 45-min session was only a single block of trials and without experimenter-imposed breaks. However, participants could take breaks between trials.

### Models

We are interested in how subjects combine sensory signals to determine whether feedback has been perturbed. The models have access to similar information: vision, proprioception, and the typical distribution of endpoint errors. They differ in how the information is combined. The *ideal observer* combines this information in a manner that maximizes the probability of being correct. A suboptimal strategy, the *comparison observer*, says *Yes* if the visual feedback and proprioceptive estimate of finger location differ sufficiently. Another suboptimal strategy, the *visual-cue-only observer,* says *Yes* only when the visual feedback indicates a large reach error and ignores proprioception.

There were two cues about the location of the reach endpoint, both of which we treated as being unidimensional: visual feedback ($\newcommand{\bialpha}{\boldsymbol{\alpha}}\newcommand{\bibeta}{\boldsymbol{\beta}}\newcommand{\bigamma}{\boldsymbol{\gamma}}\newcommand{\bidelta}{\boldsymbol{\delta}}\newcommand{\bivarepsilon}{\boldsymbol{\varepsilon}}\newcommand{\bizeta}{\boldsymbol{\zeta}}\newcommand{\bieta}{\boldsymbol{\eta}}\newcommand{\bitheta}{\boldsymbol{\theta}}\newcommand{\biiota}{\boldsymbol{\iota}}\newcommand{\bikappa}{\boldsymbol{\kappa}}\newcommand{\bilambda}{\boldsymbol{\lambda}}\newcommand{\bimu}{\boldsymbol{\mu}}\newcommand{\binu}{\boldsymbol{\nu}}\newcommand{\bixi}{\boldsymbol{\xi}}\newcommand{\biomicron}{\boldsymbol{\micron}}\newcommand{\bipi}{\boldsymbol{\pi}}\newcommand{\birho}{\boldsymbol{\rho}}\newcommand{\bisigma}{\boldsymbol{\sigma}}\newcommand{\bitau}{\boldsymbol{\tau}}\newcommand{\biupsilon}{\boldsymbol{\upsilon}}\newcommand{\biphi}{\boldsymbol{\phi}}\newcommand{\bichi}{\boldsymbol{\chi}}\newcommand{\bipsi}{\boldsymbol{\psi}}\newcommand{\biomega}{\boldsymbol{\omega}}{\hat x_v}$) and proprioceptively sensed location ($\newcommand{\bialpha}{\boldsymbol{\alpha}}\newcommand{\bibeta}{\boldsymbol{\beta}}\newcommand{\bigamma}{\boldsymbol{\gamma}}\newcommand{\bidelta}{\boldsymbol{\delta}}\newcommand{\bivarepsilon}{\boldsymbol{\varepsilon}}\newcommand{\bizeta}{\boldsymbol{\zeta}}\newcommand{\bieta}{\boldsymbol{\eta}}\newcommand{\bitheta}{\boldsymbol{\theta}}\newcommand{\biiota}{\boldsymbol{\iota}}\newcommand{\bikappa}{\boldsymbol{\kappa}}\newcommand{\bilambda}{\boldsymbol{\lambda}}\newcommand{\bimu}{\boldsymbol{\mu}}\newcommand{\binu}{\boldsymbol{\nu}}\newcommand{\bixi}{\boldsymbol{\xi}}\newcommand{\biomicron}{\boldsymbol{\micron}}\newcommand{\bipi}{\boldsymbol{\pi}}\newcommand{\birho}{\boldsymbol{\rho}}\newcommand{\bisigma}{\boldsymbol{\sigma}}\newcommand{\bitau}{\boldsymbol{\tau}}\newcommand{\biupsilon}{\boldsymbol{\upsilon}}\newcommand{\biphi}{\boldsymbol{\phi}}\newcommand{\bichi}{\boldsymbol{\chi}}\newcommand{\bipsi}{\boldsymbol{\psi}}\newcommand{\biomega}{\boldsymbol{\omega}}{\hat x_p}$). The proprioceptive signal is an unbiased estimate of the finger endpoint ($\newcommand{\bialpha}{\boldsymbol{\alpha}}\newcommand{\bibeta}{\boldsymbol{\beta}}\newcommand{\bigamma}{\boldsymbol{\gamma}}\newcommand{\bidelta}{\boldsymbol{\delta}}\newcommand{\bivarepsilon}{\boldsymbol{\varepsilon}}\newcommand{\bizeta}{\boldsymbol{\zeta}}\newcommand{\bieta}{\boldsymbol{\eta}}\newcommand{\bitheta}{\boldsymbol{\theta}}\newcommand{\biiota}{\boldsymbol{\iota}}\newcommand{\bikappa}{\boldsymbol{\kappa}}\newcommand{\bilambda}{\boldsymbol{\lambda}}\newcommand{\bimu}{\boldsymbol{\mu}}\newcommand{\binu}{\boldsymbol{\nu}}\newcommand{\bixi}{\boldsymbol{\xi}}\newcommand{\biomicron}{\boldsymbol{\micron}}\newcommand{\bipi}{\boldsymbol{\pi}}\newcommand{\birho}{\boldsymbol{\rho}}\newcommand{\bisigma}{\boldsymbol{\sigma}}\newcommand{\bitau}{\boldsymbol{\tau}}\newcommand{\biupsilon}{\boldsymbol{\upsilon}}\newcommand{\biphi}{\boldsymbol{\phi}}\newcommand{\bichi}{\boldsymbol{\chi}}\newcommand{\bipsi}{\boldsymbol{\psi}}\newcommand{\biomega}{\boldsymbol{\omega}}{x_f}$), $\newcommand{\bialpha}{\boldsymbol{\alpha}}\newcommand{\bibeta}{\boldsymbol{\beta}}\newcommand{\bigamma}{\boldsymbol{\gamma}}\newcommand{\bidelta}{\boldsymbol{\delta}}\newcommand{\bivarepsilon}{\boldsymbol{\varepsilon}}\newcommand{\bizeta}{\boldsymbol{\zeta}}\newcommand{\bieta}{\boldsymbol{\eta}}\newcommand{\bitheta}{\boldsymbol{\theta}}\newcommand{\biiota}{\boldsymbol{\iota}}\newcommand{\bikappa}{\boldsymbol{\kappa}}\newcommand{\bilambda}{\boldsymbol{\lambda}}\newcommand{\bimu}{\boldsymbol{\mu}}\newcommand{\binu}{\boldsymbol{\nu}}\newcommand{\bixi}{\boldsymbol{\xi}}\newcommand{\biomicron}{\boldsymbol{\micron}}\newcommand{\bipi}{\boldsymbol{\pi}}\newcommand{\birho}{\boldsymbol{\rho}}\newcommand{\bisigma}{\boldsymbol{\sigma}}\newcommand{\bitau}{\boldsymbol{\tau}}\newcommand{\biupsilon}{\boldsymbol{\upsilon}}\newcommand{\biphi}{\boldsymbol{\phi}}\newcommand{\bichi}{\boldsymbol{\chi}}\newcommand{\bipsi}{\boldsymbol{\psi}}\newcommand{\biomega}{\boldsymbol{\omega}}P( {{{\hat x}_p}{|}{x_f}} ) = P( {\varepsilon _p} )$, where Gaussian proprioceptive noise $\newcommand{\bialpha}{\boldsymbol{\alpha}}\newcommand{\bibeta}{\boldsymbol{\beta}}\newcommand{\bigamma}{\boldsymbol{\gamma}}\newcommand{\bidelta}{\boldsymbol{\delta}}\newcommand{\bivarepsilon}{\boldsymbol{\varepsilon}}\newcommand{\bizeta}{\boldsymbol{\zeta}}\newcommand{\bieta}{\boldsymbol{\eta}}\newcommand{\bitheta}{\boldsymbol{\theta}}\newcommand{\biiota}{\boldsymbol{\iota}}\newcommand{\bikappa}{\boldsymbol{\kappa}}\newcommand{\bilambda}{\boldsymbol{\lambda}}\newcommand{\bimu}{\boldsymbol{\mu}}\newcommand{\binu}{\boldsymbol{\nu}}\newcommand{\bixi}{\boldsymbol{\xi}}\newcommand{\biomicron}{\boldsymbol{\micron}}\newcommand{\bipi}{\boldsymbol{\pi}}\newcommand{\birho}{\boldsymbol{\rho}}\newcommand{\bisigma}{\boldsymbol{\sigma}}\newcommand{\bitau}{\boldsymbol{\tau}}\newcommand{\biupsilon}{\boldsymbol{\upsilon}}\newcommand{\biphi}{\boldsymbol{\phi}}\newcommand{\bichi}{\boldsymbol{\chi}}\newcommand{\bipsi}{\boldsymbol{\psi}}\newcommand{\biomega}{\boldsymbol{\omega}}{\varepsilon _p} = {\hat x_p} - {x_f}\sim N( {0,\sigma _p^2} )$. The visual signal is modeled as an unbiased estimate $\newcommand{\bialpha}{\boldsymbol{\alpha}}\newcommand{\bibeta}{\boldsymbol{\beta}}\newcommand{\bigamma}{\boldsymbol{\gamma}}\newcommand{\bidelta}{\boldsymbol{\delta}}\newcommand{\bivarepsilon}{\boldsymbol{\varepsilon}}\newcommand{\bizeta}{\boldsymbol{\zeta}}\newcommand{\bieta}{\boldsymbol{\eta}}\newcommand{\bitheta}{\boldsymbol{\theta}}\newcommand{\biiota}{\boldsymbol{\iota}}\newcommand{\bikappa}{\boldsymbol{\kappa}}\newcommand{\bilambda}{\boldsymbol{\lambda}}\newcommand{\bimu}{\boldsymbol{\mu}}\newcommand{\binu}{\boldsymbol{\nu}}\newcommand{\bixi}{\boldsymbol{\xi}}\newcommand{\biomicron}{\boldsymbol{\micron}}\newcommand{\bipi}{\boldsymbol{\pi}}\newcommand{\birho}{\boldsymbol{\rho}}\newcommand{\bisigma}{\boldsymbol{\sigma}}\newcommand{\bitau}{\boldsymbol{\tau}}\newcommand{\biupsilon}{\boldsymbol{\upsilon}}\newcommand{\biphi}{\boldsymbol{\phi}}\newcommand{\bichi}{\boldsymbol{\chi}}\newcommand{\bipsi}{\boldsymbol{\psi}}\newcommand{\biomega}{\boldsymbol{\omega}}{\hat x}_v$ of the visual feedback location (with perturbation $\newcommand{\bialpha}{\boldsymbol{\alpha}}\newcommand{\bibeta}{\boldsymbol{\beta}}\newcommand{\bigamma}{\boldsymbol{\gamma}}\newcommand{\bidelta}{\boldsymbol{\delta}}\newcommand{\bivarepsilon}{\boldsymbol{\varepsilon}}\newcommand{\bizeta}{\boldsymbol{\zeta}}\newcommand{\bieta}{\boldsymbol{\eta}}\newcommand{\bitheta}{\boldsymbol{\theta}}\newcommand{\biiota}{\boldsymbol{\iota}}\newcommand{\bikappa}{\boldsymbol{\kappa}}\newcommand{\bilambda}{\boldsymbol{\lambda}}\newcommand{\bimu}{\boldsymbol{\mu}}\newcommand{\binu}{\boldsymbol{\nu}}\newcommand{\bixi}{\boldsymbol{\xi}}\newcommand{\biomicron}{\boldsymbol{\micron}}\newcommand{\bipi}{\boldsymbol{\pi}}\newcommand{\birho}{\boldsymbol{\rho}}\newcommand{\bisigma}{\boldsymbol{\sigma}}\newcommand{\bitau}{\boldsymbol{\tau}}\newcommand{\biupsilon}{\boldsymbol{\upsilon}}\newcommand{\biphi}{\boldsymbol{\phi}}\newcommand{\bichi}{\boldsymbol{\chi}}\newcommand{\bipsi}{\boldsymbol{\psi}}\newcommand{\biomega}{\boldsymbol{\omega}}{\rm{\Delta }}{x_v}$), $\newcommand{\bialpha}{\boldsymbol{\alpha}}\newcommand{\bibeta}{\boldsymbol{\beta}}\newcommand{\bigamma}{\boldsymbol{\gamma}}\newcommand{\bidelta}{\boldsymbol{\delta}}\newcommand{\bivarepsilon}{\boldsymbol{\varepsilon}}\newcommand{\bizeta}{\boldsymbol{\zeta}}\newcommand{\bieta}{\boldsymbol{\eta}}\newcommand{\bitheta}{\boldsymbol{\theta}}\newcommand{\biiota}{\boldsymbol{\iota}}\newcommand{\bikappa}{\boldsymbol{\kappa}}\newcommand{\bilambda}{\boldsymbol{\lambda}}\newcommand{\bimu}{\boldsymbol{\mu}}\newcommand{\binu}{\boldsymbol{\nu}}\newcommand{\bixi}{\boldsymbol{\xi}}\newcommand{\biomicron}{\boldsymbol{\micron}}\newcommand{\bipi}{\boldsymbol{\pi}}\newcommand{\birho}{\boldsymbol{\rho}}\newcommand{\bisigma}{\boldsymbol{\sigma}}\newcommand{\bitau}{\boldsymbol{\tau}}\newcommand{\biupsilon}{\boldsymbol{\upsilon}}\newcommand{\biphi}{\boldsymbol{\phi}}\newcommand{\bichi}{\boldsymbol{\chi}}\newcommand{\bipsi}{\boldsymbol{\psi}}\newcommand{\biomega}{\boldsymbol{\omega}}{x_v} = {x_f} + {\rm{\Delta }}{x_v}$, with $\newcommand{\bialpha}{\boldsymbol{\alpha}}\newcommand{\bibeta}{\boldsymbol{\beta}}\newcommand{\bigamma}{\boldsymbol{\gamma}}\newcommand{\bidelta}{\boldsymbol{\delta}}\newcommand{\bivarepsilon}{\boldsymbol{\varepsilon}}\newcommand{\bizeta}{\boldsymbol{\zeta}}\newcommand{\bieta}{\boldsymbol{\eta}}\newcommand{\bitheta}{\boldsymbol{\theta}}\newcommand{\biiota}{\boldsymbol{\iota}}\newcommand{\bikappa}{\boldsymbol{\kappa}}\newcommand{\bilambda}{\boldsymbol{\lambda}}\newcommand{\bimu}{\boldsymbol{\mu}}\newcommand{\binu}{\boldsymbol{\nu}}\newcommand{\bixi}{\boldsymbol{\xi}}\newcommand{\biomicron}{\boldsymbol{\micron}}\newcommand{\bipi}{\boldsymbol{\pi}}\newcommand{\birho}{\boldsymbol{\rho}}\newcommand{\bisigma}{\boldsymbol{\sigma}}\newcommand{\bitau}{\boldsymbol{\tau}}\newcommand{\biupsilon}{\boldsymbol{\upsilon}}\newcommand{\biphi}{\boldsymbol{\phi}}\newcommand{\bichi}{\boldsymbol{\chi}}\newcommand{\bipsi}{\boldsymbol{\psi}}\newcommand{\biomega}{\boldsymbol{\omega}}P( {{{\hat x}_v}{|}{x_f},{\rm{\Delta }}{x_v}} ) = P\left( {\varepsilon _v} \right)$, where Gaussian visual noise $\newcommand{\bialpha}{\boldsymbol{\alpha}}\newcommand{\bibeta}{\boldsymbol{\beta}}\newcommand{\bigamma}{\boldsymbol{\gamma}}\newcommand{\bidelta}{\boldsymbol{\delta}}\newcommand{\bivarepsilon}{\boldsymbol{\varepsilon}}\newcommand{\bizeta}{\boldsymbol{\zeta}}\newcommand{\bieta}{\boldsymbol{\eta}}\newcommand{\bitheta}{\boldsymbol{\theta}}\newcommand{\biiota}{\boldsymbol{\iota}}\newcommand{\bikappa}{\boldsymbol{\kappa}}\newcommand{\bilambda}{\boldsymbol{\lambda}}\newcommand{\bimu}{\boldsymbol{\mu}}\newcommand{\binu}{\boldsymbol{\nu}}\newcommand{\bixi}{\boldsymbol{\xi}}\newcommand{\biomicron}{\boldsymbol{\micron}}\newcommand{\bipi}{\boldsymbol{\pi}}\newcommand{\birho}{\boldsymbol{\rho}}\newcommand{\bisigma}{\boldsymbol{\sigma}}\newcommand{\bitau}{\boldsymbol{\tau}}\newcommand{\biupsilon}{\boldsymbol{\upsilon}}\newcommand{\biphi}{\boldsymbol{\phi}}\newcommand{\bichi}{\boldsymbol{\chi}}\newcommand{\bipsi}{\boldsymbol{\psi}}\newcommand{\biomega}{\boldsymbol{\omega}}{\varepsilon _v} = {\hat x_v} - ( {{x_f} + {\rm{\Delta }}{x_v}} )\sim N( {0,\sigma _v^2} )$. We compared three potential models of subjects' behavior. Of course, we do not have direct access to subjects' proprioceptive signal. Rather, we looked at how subjects' decisions were affected by the size of the perturbation and the reach endpoint position. Reach endpoints appear to be normally distributed (via a q-q plot), although hypometric. Hypometria and a constant rotational error during the gain and direction sessions can be accounted for by including a bias term $\newcommand{\bialpha}{\boldsymbol{\alpha}}\newcommand{\bibeta}{\boldsymbol{\beta}}\newcommand{\bigamma}{\boldsymbol{\gamma}}\newcommand{\bidelta}{\boldsymbol{\delta}}\newcommand{\bivarepsilon}{\boldsymbol{\varepsilon}}\newcommand{\bizeta}{\boldsymbol{\zeta}}\newcommand{\bieta}{\boldsymbol{\eta}}\newcommand{\bitheta}{\boldsymbol{\theta}}\newcommand{\biiota}{\boldsymbol{\iota}}\newcommand{\bikappa}{\boldsymbol{\kappa}}\newcommand{\bilambda}{\boldsymbol{\lambda}}\newcommand{\bimu}{\boldsymbol{\mu}}\newcommand{\binu}{\boldsymbol{\nu}}\newcommand{\bixi}{\boldsymbol{\xi}}\newcommand{\biomicron}{\boldsymbol{\micron}}\newcommand{\bipi}{\boldsymbol{\pi}}\newcommand{\birho}{\boldsymbol{\rho}}\newcommand{\bisigma}{\boldsymbol{\sigma}}\newcommand{\bitau}{\boldsymbol{\tau}}\newcommand{\biupsilon}{\boldsymbol{\upsilon}}\newcommand{\biphi}{\boldsymbol{\phi}}\newcommand{\bichi}{\boldsymbol{\chi}}\newcommand{\bipsi}{\boldsymbol{\psi}}\newcommand{\biomega}{\boldsymbol{\omega}}b$ in the average reach to the target position ($\newcommand{\bialpha}{\boldsymbol{\alpha}}\newcommand{\bibeta}{\boldsymbol{\beta}}\newcommand{\bigamma}{\boldsymbol{\gamma}}\newcommand{\bidelta}{\boldsymbol{\delta}}\newcommand{\bivarepsilon}{\boldsymbol{\varepsilon}}\newcommand{\bizeta}{\boldsymbol{\zeta}}\newcommand{\bieta}{\boldsymbol{\eta}}\newcommand{\bitheta}{\boldsymbol{\theta}}\newcommand{\biiota}{\boldsymbol{\iota}}\newcommand{\bikappa}{\boldsymbol{\kappa}}\newcommand{\bilambda}{\boldsymbol{\lambda}}\newcommand{\bimu}{\boldsymbol{\mu}}\newcommand{\binu}{\boldsymbol{\nu}}\newcommand{\bixi}{\boldsymbol{\xi}}\newcommand{\biomicron}{\boldsymbol{\micron}}\newcommand{\bipi}{\boldsymbol{\pi}}\newcommand{\birho}{\boldsymbol{\rho}}\newcommand{\bisigma}{\boldsymbol{\sigma}}\newcommand{\bitau}{\boldsymbol{\tau}}\newcommand{\biupsilon}{\boldsymbol{\upsilon}}\newcommand{\biphi}{\boldsymbol{\phi}}\newcommand{\bichi}{\boldsymbol{\chi}}\newcommand{\bipsi}{\boldsymbol{\psi}}\newcommand{\biomega}{\boldsymbol{\omega}}{x_t}$, which we define here as 0), and we assume the observer takes this bias into account. Therefore, $\newcommand{\bialpha}{\boldsymbol{\alpha}}\newcommand{\bibeta}{\boldsymbol{\beta}}\newcommand{\bigamma}{\boldsymbol{\gamma}}\newcommand{\bidelta}{\boldsymbol{\delta}}\newcommand{\bivarepsilon}{\boldsymbol{\varepsilon}}\newcommand{\bizeta}{\boldsymbol{\zeta}}\newcommand{\bieta}{\boldsymbol{\eta}}\newcommand{\bitheta}{\boldsymbol{\theta}}\newcommand{\biiota}{\boldsymbol{\iota}}\newcommand{\bikappa}{\boldsymbol{\kappa}}\newcommand{\bilambda}{\boldsymbol{\lambda}}\newcommand{\bimu}{\boldsymbol{\mu}}\newcommand{\binu}{\boldsymbol{\nu}}\newcommand{\bixi}{\boldsymbol{\xi}}\newcommand{\biomicron}{\boldsymbol{\micron}}\newcommand{\bipi}{\boldsymbol{\pi}}\newcommand{\birho}{\boldsymbol{\rho}}\newcommand{\bisigma}{\boldsymbol{\sigma}}\newcommand{\bitau}{\boldsymbol{\tau}}\newcommand{\biupsilon}{\boldsymbol{\upsilon}}\newcommand{\biphi}{\boldsymbol{\phi}}\newcommand{\bichi}{\boldsymbol{\chi}}\newcommand{\bipsi}{\boldsymbol{\psi}}\newcommand{\biomega}{\boldsymbol{\omega}}P\left( {x_f} \right) = P\left( {\varepsilon _f} \right)$, where $\newcommand{\bialpha}{\boldsymbol{\alpha}}\newcommand{\bibeta}{\boldsymbol{\beta}}\newcommand{\bigamma}{\boldsymbol{\gamma}}\newcommand{\bidelta}{\boldsymbol{\delta}}\newcommand{\bivarepsilon}{\boldsymbol{\varepsilon}}\newcommand{\bizeta}{\boldsymbol{\zeta}}\newcommand{\bieta}{\boldsymbol{\eta}}\newcommand{\bitheta}{\boldsymbol{\theta}}\newcommand{\biiota}{\boldsymbol{\iota}}\newcommand{\bikappa}{\boldsymbol{\kappa}}\newcommand{\bilambda}{\boldsymbol{\lambda}}\newcommand{\bimu}{\boldsymbol{\mu}}\newcommand{\binu}{\boldsymbol{\nu}}\newcommand{\bixi}{\boldsymbol{\xi}}\newcommand{\biomicron}{\boldsymbol{\micron}}\newcommand{\bipi}{\boldsymbol{\pi}}\newcommand{\birho}{\boldsymbol{\rho}}\newcommand{\bisigma}{\boldsymbol{\sigma}}\newcommand{\bitau}{\boldsymbol{\tau}}\newcommand{\biupsilon}{\boldsymbol{\upsilon}}\newcommand{\biphi}{\boldsymbol{\phi}}\newcommand{\bichi}{\boldsymbol{\chi}}\newcommand{\bipsi}{\boldsymbol{\psi}}\newcommand{\biomega}{\boldsymbol{\omega}}{\varepsilon _f} = {x_f}\sim N( {b,\sigma _f^2} )$. The data were normalized by the standard deviation of the motor noise from each session separately, so normalized motor errors over all sessions have $\newcommand{\bialpha}{\boldsymbol{\alpha}}\newcommand{\bibeta}{\boldsymbol{\beta}}\newcommand{\bigamma}{\boldsymbol{\gamma}}\newcommand{\bidelta}{\boldsymbol{\delta}}\newcommand{\bivarepsilon}{\boldsymbol{\varepsilon}}\newcommand{\bizeta}{\boldsymbol{\zeta}}\newcommand{\bieta}{\boldsymbol{\eta}}\newcommand{\bitheta}{\boldsymbol{\theta}}\newcommand{\biiota}{\boldsymbol{\iota}}\newcommand{\bikappa}{\boldsymbol{\kappa}}\newcommand{\bilambda}{\boldsymbol{\lambda}}\newcommand{\bimu}{\boldsymbol{\mu}}\newcommand{\binu}{\boldsymbol{\nu}}\newcommand{\bixi}{\boldsymbol{\xi}}\newcommand{\biomicron}{\boldsymbol{\micron}}\newcommand{\bipi}{\boldsymbol{\pi}}\newcommand{\birho}{\boldsymbol{\rho}}\newcommand{\bisigma}{\boldsymbol{\sigma}}\newcommand{\bitau}{\boldsymbol{\tau}}\newcommand{\biupsilon}{\boldsymbol{\upsilon}}\newcommand{\biphi}{\boldsymbol{\phi}}\newcommand{\bichi}{\boldsymbol{\chi}}\newcommand{\bipsi}{\boldsymbol{\psi}}\newcommand{\biomega}{\boldsymbol{\omega}}{\sigma _f} \approx 1$. Similar to the observer's estimate of the visual feedback location, the observer's estimate of the target location ($\newcommand{\bialpha}{\boldsymbol{\alpha}}\newcommand{\bibeta}{\boldsymbol{\beta}}\newcommand{\bigamma}{\boldsymbol{\gamma}}\newcommand{\bidelta}{\boldsymbol{\delta}}\newcommand{\bivarepsilon}{\boldsymbol{\varepsilon}}\newcommand{\bizeta}{\boldsymbol{\zeta}}\newcommand{\bieta}{\boldsymbol{\eta}}\newcommand{\bitheta}{\boldsymbol{\theta}}\newcommand{\biiota}{\boldsymbol{\iota}}\newcommand{\bikappa}{\boldsymbol{\kappa}}\newcommand{\bilambda}{\boldsymbol{\lambda}}\newcommand{\bimu}{\boldsymbol{\mu}}\newcommand{\binu}{\boldsymbol{\nu}}\newcommand{\bixi}{\boldsymbol{\xi}}\newcommand{\biomicron}{\boldsymbol{\micron}}\newcommand{\bipi}{\boldsymbol{\pi}}\newcommand{\birho}{\boldsymbol{\rho}}\newcommand{\bisigma}{\boldsymbol{\sigma}}\newcommand{\bitau}{\boldsymbol{\tau}}\newcommand{\biupsilon}{\boldsymbol{\upsilon}}\newcommand{\biphi}{\boldsymbol{\phi}}\newcommand{\bichi}{\boldsymbol{\chi}}\newcommand{\bipsi}{\boldsymbol{\psi}}\newcommand{\biomega}{\boldsymbol{\omega}}{\hat x_t}$) is also corrupted by visual noise, so that $\newcommand{\bialpha}{\boldsymbol{\alpha}}\newcommand{\bibeta}{\boldsymbol{\beta}}\newcommand{\bigamma}{\boldsymbol{\gamma}}\newcommand{\bidelta}{\boldsymbol{\delta}}\newcommand{\bivarepsilon}{\boldsymbol{\varepsilon}}\newcommand{\bizeta}{\boldsymbol{\zeta}}\newcommand{\bieta}{\boldsymbol{\eta}}\newcommand{\bitheta}{\boldsymbol{\theta}}\newcommand{\biiota}{\boldsymbol{\iota}}\newcommand{\bikappa}{\boldsymbol{\kappa}}\newcommand{\bilambda}{\boldsymbol{\lambda}}\newcommand{\bimu}{\boldsymbol{\mu}}\newcommand{\binu}{\boldsymbol{\nu}}\newcommand{\bixi}{\boldsymbol{\xi}}\newcommand{\biomicron}{\boldsymbol{\micron}}\newcommand{\bipi}{\boldsymbol{\pi}}\newcommand{\birho}{\boldsymbol{\rho}}\newcommand{\bisigma}{\boldsymbol{\sigma}}\newcommand{\bitau}{\boldsymbol{\tau}}\newcommand{\biupsilon}{\boldsymbol{\upsilon}}\newcommand{\biphi}{\boldsymbol{\phi}}\newcommand{\bichi}{\boldsymbol{\chi}}\newcommand{\bipsi}{\boldsymbol{\psi}}\newcommand{\biomega}{\boldsymbol{\omega}}P\left( {{{\hat x}_t}} \right) = P\left( {\varepsilon _v} \right)$. We performed a formal model comparison to determine which strategy participants used.

#### Ideal observer

The *ideal observer* computes and compares the probability of a perturbation $\newcommand{\bialpha}{\boldsymbol{\alpha}}\newcommand{\bibeta}{\boldsymbol{\beta}}\newcommand{\bigamma}{\boldsymbol{\gamma}}\newcommand{\bidelta}{\boldsymbol{\delta}}\newcommand{\bivarepsilon}{\boldsymbol{\varepsilon}}\newcommand{\bizeta}{\boldsymbol{\zeta}}\newcommand{\bieta}{\boldsymbol{\eta}}\newcommand{\bitheta}{\boldsymbol{\theta}}\newcommand{\biiota}{\boldsymbol{\iota}}\newcommand{\bikappa}{\boldsymbol{\kappa}}\newcommand{\bilambda}{\boldsymbol{\lambda}}\newcommand{\bimu}{\boldsymbol{\mu}}\newcommand{\binu}{\boldsymbol{\nu}}\newcommand{\bixi}{\boldsymbol{\xi}}\newcommand{\biomicron}{\boldsymbol{\micron}}\newcommand{\bipi}{\boldsymbol{\pi}}\newcommand{\birho}{\boldsymbol{\rho}}\newcommand{\bisigma}{\boldsymbol{\sigma}}\newcommand{\bitau}{\boldsymbol{\tau}}\newcommand{\biupsilon}{\boldsymbol{\upsilon}}\newcommand{\biphi}{\boldsymbol{\phi}}\newcommand{\bichi}{\boldsymbol{\chi}}\newcommand{\bipsi}{\boldsymbol{\psi}}\newcommand{\biomega}{\boldsymbol{\omega}}\left( {{\rm{\Delta }}{x_v} \ne 0} \right)$ and no perturbation ($\newcommand{\bialpha}{\boldsymbol{\alpha}}\newcommand{\bibeta}{\boldsymbol{\beta}}\newcommand{\bigamma}{\boldsymbol{\gamma}}\newcommand{\bidelta}{\boldsymbol{\delta}}\newcommand{\bivarepsilon}{\boldsymbol{\varepsilon}}\newcommand{\bizeta}{\boldsymbol{\zeta}}\newcommand{\bieta}{\boldsymbol{\eta}}\newcommand{\bitheta}{\boldsymbol{\theta}}\newcommand{\biiota}{\boldsymbol{\iota}}\newcommand{\bikappa}{\boldsymbol{\kappa}}\newcommand{\bilambda}{\boldsymbol{\lambda}}\newcommand{\bimu}{\boldsymbol{\mu}}\newcommand{\binu}{\boldsymbol{\nu}}\newcommand{\bixi}{\boldsymbol{\xi}}\newcommand{\biomicron}{\boldsymbol{\micron}}\newcommand{\bipi}{\boldsymbol{\pi}}\newcommand{\birho}{\boldsymbol{\rho}}\newcommand{\bisigma}{\boldsymbol{\sigma}}\newcommand{\bitau}{\boldsymbol{\tau}}\newcommand{\biupsilon}{\boldsymbol{\upsilon}}\newcommand{\biphi}{\boldsymbol{\phi}}\newcommand{\bichi}{\boldsymbol{\chi}}\newcommand{\bipsi}{\boldsymbol{\psi}}\newcommand{\biomega}{\boldsymbol{\omega}}{\rm{\Delta }}{x_v} = 0$) given the sensory signals. The response is based on whichever was greater. Given that the probability of a perturbation was 0.5 in our experiment, the ideal observer detects a perturbation when the likelihood of the current visual and proprioceptive sensory signals is greater on the assumption of a perturbation than on the assumption of no perturbation:

$$\newcommand{\bialpha}{\boldsymbol{\alpha}}\newcommand{\bibeta}{\boldsymbol{\beta}}\newcommand{\bigamma}{\boldsymbol{\gamma}}\newcommand{\bidelta}{\boldsymbol{\delta}}\newcommand{\bivarepsilon}{\boldsymbol{\varepsilon}}\newcommand{\bizeta}{\boldsymbol{\zeta}}\newcommand{\bieta}{\boldsymbol{\eta}}\newcommand{\bitheta}{\boldsymbol{\theta}}\newcommand{\biiota}{\boldsymbol{\iota}}\newcommand{\bikappa}{\boldsymbol{\kappa}}\newcommand{\bilambda}{\boldsymbol{\lambda}}\newcommand{\bimu}{\boldsymbol{\mu}}\newcommand{\binu}{\boldsymbol{\nu}}\newcommand{\bixi}{\boldsymbol{\xi}}\newcommand{\biomicron}{\boldsymbol{\micron}}\newcommand{\bipi}{\boldsymbol{\pi}}\newcommand{\birho}{\boldsymbol{\rho}}\newcommand{\bisigma}{\boldsymbol{\sigma}}\newcommand{\bitau}{\boldsymbol{\tau}}\newcommand{\biupsilon}{\boldsymbol{\upsilon}}\newcommand{\biphi}{\boldsymbol{\phi}}\newcommand{\bichi}{\boldsymbol{\chi}}\newcommand{\bipsi}{\boldsymbol{\psi}}\newcommand{\biomega}{\boldsymbol{\omega}}P\left( {{{\hat x}_p},{{\hat x}_v}{\rm{|\Delta }}{x_v} \ne 0} \right) \gt P\left( {{{\hat x}_p},{{\hat x}_v}{\rm{|\Delta }}{x_v} = 0} \right){\rm {.}}$$

The likelihood of the no-perturbation hypothesis is computed by integrating over all possible finger endpoint positions:

$$\newcommand{\bialpha}{\boldsymbol{\alpha}}\newcommand{\bibeta}{\boldsymbol{\beta}}\newcommand{\bigamma}{\boldsymbol{\gamma}}\newcommand{\bidelta}{\boldsymbol{\delta}}\newcommand{\bivarepsilon}{\boldsymbol{\varepsilon}}\newcommand{\bizeta}{\boldsymbol{\zeta}}\newcommand{\bieta}{\boldsymbol{\eta}}\newcommand{\bitheta}{\boldsymbol{\theta}}\newcommand{\biiota}{\boldsymbol{\iota}}\newcommand{\bikappa}{\boldsymbol{\kappa}}\newcommand{\bilambda}{\boldsymbol{\lambda}}\newcommand{\bimu}{\boldsymbol{\mu}}\newcommand{\binu}{\boldsymbol{\nu}}\newcommand{\bixi}{\boldsymbol{\xi}}\newcommand{\biomicron}{\boldsymbol{\micron}}\newcommand{\bipi}{\boldsymbol{\pi}}\newcommand{\birho}{\boldsymbol{\rho}}\newcommand{\bisigma}{\boldsymbol{\sigma}}\newcommand{\bitau}{\boldsymbol{\tau}}\newcommand{\biupsilon}{\boldsymbol{\upsilon}}\newcommand{\biphi}{\boldsymbol{\phi}}\newcommand{\bichi}{\boldsymbol{\chi}}\newcommand{\bipsi}{\boldsymbol{\psi}}\newcommand{\biomega}{\boldsymbol{\omega}}P\left( {{{\hat x}_p},{{\hat x}_v}{\rm{|\Delta }}{x_v} = 0} \right) = \mathop \int \nolimits P \left( {{{\hat x}_v}{|}{x_f},{\rm{\Delta }}{x_v} = 0} \right)P\left( {{{\hat x}_p}{|}{x_f}} \right)P\left( {{x_f}{|}{x_t}} \right)d{x_f}{\rm {.}}$$

$\newcommand{\bialpha}{\boldsymbol{\alpha}}\newcommand{\bibeta}{\boldsymbol{\beta}}\newcommand{\bigamma}{\boldsymbol{\gamma}}\newcommand{\bidelta}{\boldsymbol{\delta}}\newcommand{\bivarepsilon}{\boldsymbol{\varepsilon}}\newcommand{\bizeta}{\boldsymbol{\zeta}}\newcommand{\bieta}{\boldsymbol{\eta}}\newcommand{\bitheta}{\boldsymbol{\theta}}\newcommand{\biiota}{\boldsymbol{\iota}}\newcommand{\bikappa}{\boldsymbol{\kappa}}\newcommand{\bilambda}{\boldsymbol{\lambda}}\newcommand{\bimu}{\boldsymbol{\mu}}\newcommand{\binu}{\boldsymbol{\nu}}\newcommand{\bixi}{\boldsymbol{\xi}}\newcommand{\biomicron}{\boldsymbol{\micron}}\newcommand{\bipi}{\boldsymbol{\pi}}\newcommand{\birho}{\boldsymbol{\rho}}\newcommand{\bisigma}{\boldsymbol{\sigma}}\newcommand{\bitau}{\boldsymbol{\tau}}\newcommand{\biupsilon}{\boldsymbol{\upsilon}}\newcommand{\biphi}{\boldsymbol{\phi}}\newcommand{\bichi}{\boldsymbol{\chi}}\newcommand{\bipsi}{\boldsymbol{\psi}}\newcommand{\biomega}{\boldsymbol{\omega}}P\left( {{x_f}{|}{x_t}} \right)$ is the observer's knowledge of the distribution of finger endpoints, and introduces the bias, $\newcommand{\bialpha}{\boldsymbol{\alpha}}\newcommand{\bibeta}{\boldsymbol{\beta}}\newcommand{\bigamma}{\boldsymbol{\gamma}}\newcommand{\bidelta}{\boldsymbol{\delta}}\newcommand{\bivarepsilon}{\boldsymbol{\varepsilon}}\newcommand{\bizeta}{\boldsymbol{\zeta}}\newcommand{\bieta}{\boldsymbol{\eta}}\newcommand{\bitheta}{\boldsymbol{\theta}}\newcommand{\biiota}{\boldsymbol{\iota}}\newcommand{\bikappa}{\boldsymbol{\kappa}}\newcommand{\bilambda}{\boldsymbol{\lambda}}\newcommand{\bimu}{\boldsymbol{\mu}}\newcommand{\binu}{\boldsymbol{\nu}}\newcommand{\bixi}{\boldsymbol{\xi}}\newcommand{\biomicron}{\boldsymbol{\micron}}\newcommand{\bipi}{\boldsymbol{\pi}}\newcommand{\birho}{\boldsymbol{\rho}}\newcommand{\bisigma}{\boldsymbol{\sigma}}\newcommand{\bitau}{\boldsymbol{\tau}}\newcommand{\biupsilon}{\boldsymbol{\upsilon}}\newcommand{\biphi}{\boldsymbol{\phi}}\newcommand{\bichi}{\boldsymbol{\chi}}\newcommand{\bipsi}{\boldsymbol{\psi}}\newcommand{\biomega}{\boldsymbol{\omega}}b$, the subject's expected mean endpoint. Substituting for each probability, we have

$$\newcommand{\bialpha}{\boldsymbol{\alpha}}\newcommand{\bibeta}{\boldsymbol{\beta}}\newcommand{\bigamma}{\boldsymbol{\gamma}}\newcommand{\bidelta}{\boldsymbol{\delta}}\newcommand{\bivarepsilon}{\boldsymbol{\varepsilon}}\newcommand{\bizeta}{\boldsymbol{\zeta}}\newcommand{\bieta}{\boldsymbol{\eta}}\newcommand{\bitheta}{\boldsymbol{\theta}}\newcommand{\biiota}{\boldsymbol{\iota}}\newcommand{\bikappa}{\boldsymbol{\kappa}}\newcommand{\bilambda}{\boldsymbol{\lambda}}\newcommand{\bimu}{\boldsymbol{\mu}}\newcommand{\binu}{\boldsymbol{\nu}}\newcommand{\bixi}{\boldsymbol{\xi}}\newcommand{\biomicron}{\boldsymbol{\micron}}\newcommand{\bipi}{\boldsymbol{\pi}}\newcommand{\birho}{\boldsymbol{\rho}}\newcommand{\bisigma}{\boldsymbol{\sigma}}\newcommand{\bitau}{\boldsymbol{\tau}}\newcommand{\biupsilon}{\boldsymbol{\upsilon}}\newcommand{\biphi}{\boldsymbol{\phi}}\newcommand{\bichi}{\boldsymbol{\chi}}\newcommand{\bipsi}{\boldsymbol{\psi}}\newcommand{\biomega}{\boldsymbol{\omega}}P\left( {{{\hat x}_p},{{\hat x}_v}{\rm{|\Delta }}{x_v} = 0} \right) = \mathop \int \nolimits {1 \over {\sqrt {2\pi } {\sigma _v}}}exp\left( { - {{\left( {{{\hat x}_v} - {x_f}} \right)}^2}/2\sigma _v^2} \right) \times {1 \over {\sqrt {2\pi } {\sigma _p}}}exp\left( { - {{\left( {{{\hat x}_p} - {x_f}} \right)}^2}/2\sigma _p^2} \right) \times {1 \over {\sqrt {2\pi } {\sigma _f}}}exp\left( { - {{\left( {{x_f} - \left( {{x_t} - b} \right)} \right)}^2}/2\sigma _f^2} \right)\,d{x_f}.$$

The likelihood of the perturbation hypothesis is slightly more complicated, as the observer also needs to integrate over possible values of the perturbation.

$$\newcommand{\bialpha}{\boldsymbol{\alpha}}\newcommand{\bibeta}{\boldsymbol{\beta}}\newcommand{\bigamma}{\boldsymbol{\gamma}}\newcommand{\bidelta}{\boldsymbol{\delta}}\newcommand{\bivarepsilon}{\boldsymbol{\varepsilon}}\newcommand{\bizeta}{\boldsymbol{\zeta}}\newcommand{\bieta}{\boldsymbol{\eta}}\newcommand{\bitheta}{\boldsymbol{\theta}}\newcommand{\biiota}{\boldsymbol{\iota}}\newcommand{\bikappa}{\boldsymbol{\kappa}}\newcommand{\bilambda}{\boldsymbol{\lambda}}\newcommand{\bimu}{\boldsymbol{\mu}}\newcommand{\binu}{\boldsymbol{\nu}}\newcommand{\bixi}{\boldsymbol{\xi}}\newcommand{\biomicron}{\boldsymbol{\micron}}\newcommand{\bipi}{\boldsymbol{\pi}}\newcommand{\birho}{\boldsymbol{\rho}}\newcommand{\bisigma}{\boldsymbol{\sigma}}\newcommand{\bitau}{\boldsymbol{\tau}}\newcommand{\biupsilon}{\boldsymbol{\upsilon}}\newcommand{\biphi}{\boldsymbol{\phi}}\newcommand{\bichi}{\boldsymbol{\chi}}\newcommand{\bipsi}{\boldsymbol{\psi}}\newcommand{\biomega}{\boldsymbol{\omega}}P\left( {{{\hat x}_p},{{\hat x}_v}{\rm{|\Delta }}{x_v} \ne 0} \right) = \mathop \int\!\!\!\!\!\int \nolimits\! \! P \left( {{{\hat x}_v}{|}{x_v}} \right)P\left( {{{\hat x}_p}{|}{x_f}} \right)P\left( {{\rm{\Delta }}{x_v}} \right)P\left( {{x_f}{|}{x_t}} \right)d{\rm{\Delta }}{x_v}\,d{x_f}{\rm {.}}$$

For the probability of the magnitude of the perturbation, $\newcommand{\bialpha}{\boldsymbol{\alpha}}\newcommand{\bibeta}{\boldsymbol{\beta}}\newcommand{\bigamma}{\boldsymbol{\gamma}}\newcommand{\bidelta}{\boldsymbol{\delta}}\newcommand{\bivarepsilon}{\boldsymbol{\varepsilon}}\newcommand{\bizeta}{\boldsymbol{\zeta}}\newcommand{\bieta}{\boldsymbol{\eta}}\newcommand{\bitheta}{\boldsymbol{\theta}}\newcommand{\biiota}{\boldsymbol{\iota}}\newcommand{\bikappa}{\boldsymbol{\kappa}}\newcommand{\bilambda}{\boldsymbol{\lambda}}\newcommand{\bimu}{\boldsymbol{\mu}}\newcommand{\binu}{\boldsymbol{\nu}}\newcommand{\bixi}{\boldsymbol{\xi}}\newcommand{\biomicron}{\boldsymbol{\micron}}\newcommand{\bipi}{\boldsymbol{\pi}}\newcommand{\birho}{\boldsymbol{\rho}}\newcommand{\bisigma}{\boldsymbol{\sigma}}\newcommand{\bitau}{\boldsymbol{\tau}}\newcommand{\biupsilon}{\boldsymbol{\upsilon}}\newcommand{\biphi}{\boldsymbol{\phi}}\newcommand{\bichi}{\boldsymbol{\chi}}\newcommand{\bipsi}{\boldsymbol{\psi}}\newcommand{\biomega}{\boldsymbol{\omega}}P\left( {{\rm{\Delta }}{x_v}} \right)$, we simplified by assuming the observer believes the perturbation values to be normally distributed. We used a standard deviation *σ_pert_* equal to the true *SD* of the experimental values, even though we used the method of constant stimuli with 50% catch trials, so that $\newcommand{\bialpha}{\boldsymbol{\alpha}}\newcommand{\bibeta}{\boldsymbol{\beta}}\newcommand{\bigamma}{\boldsymbol{\gamma}}\newcommand{\bidelta}{\boldsymbol{\delta}}\newcommand{\bivarepsilon}{\boldsymbol{\varepsilon}}\newcommand{\bizeta}{\boldsymbol{\zeta}}\newcommand{\bieta}{\boldsymbol{\eta}}\newcommand{\bitheta}{\boldsymbol{\theta}}\newcommand{\biiota}{\boldsymbol{\iota}}\newcommand{\bikappa}{\boldsymbol{\kappa}}\newcommand{\bilambda}{\boldsymbol{\lambda}}\newcommand{\bimu}{\boldsymbol{\mu}}\newcommand{\binu}{\boldsymbol{\nu}}\newcommand{\bixi}{\boldsymbol{\xi}}\newcommand{\biomicron}{\boldsymbol{\micron}}\newcommand{\bipi}{\boldsymbol{\pi}}\newcommand{\birho}{\boldsymbol{\rho}}\newcommand{\bisigma}{\boldsymbol{\sigma}}\newcommand{\bitau}{\boldsymbol{\tau}}\newcommand{\biupsilon}{\boldsymbol{\upsilon}}\newcommand{\biphi}{\boldsymbol{\phi}}\newcommand{\bichi}{\boldsymbol{\chi}}\newcommand{\bipsi}{\boldsymbol{\psi}}\newcommand{\biomega}{\boldsymbol{\omega}}{\rm{\Delta }}{x_v}\sim N\left( {0,{\sigma _{pert}^2}} \right)$. Taken together:

$$\newcommand{\bialpha}{\boldsymbol{\alpha}}\newcommand{\bibeta}{\boldsymbol{\beta}}\newcommand{\bigamma}{\boldsymbol{\gamma}}\newcommand{\bidelta}{\boldsymbol{\delta}}\newcommand{\bivarepsilon}{\boldsymbol{\varepsilon}}\newcommand{\bizeta}{\boldsymbol{\zeta}}\newcommand{\bieta}{\boldsymbol{\eta}}\newcommand{\bitheta}{\boldsymbol{\theta}}\newcommand{\biiota}{\boldsymbol{\iota}}\newcommand{\bikappa}{\boldsymbol{\kappa}}\newcommand{\bilambda}{\boldsymbol{\lambda}}\newcommand{\bimu}{\boldsymbol{\mu}}\newcommand{\binu}{\boldsymbol{\nu}}\newcommand{\bixi}{\boldsymbol{\xi}}\newcommand{\biomicron}{\boldsymbol{\micron}}\newcommand{\bipi}{\boldsymbol{\pi}}\newcommand{\birho}{\boldsymbol{\rho}}\newcommand{\bisigma}{\boldsymbol{\sigma}}\newcommand{\bitau}{\boldsymbol{\tau}}\newcommand{\biupsilon}{\boldsymbol{\upsilon}}\newcommand{\biphi}{\boldsymbol{\phi}}\newcommand{\bichi}{\boldsymbol{\chi}}\newcommand{\bipsi}{\boldsymbol{\psi}}\newcommand{\biomega}{\boldsymbol{\omega}}P\left( {{{\hat x}_p},{{\hat x}_v}{\rm{|\Delta }}{x_v} \ne 0} \right) = \mathop \int\!\!\!\!\!\int \nolimits {1 \over {\sqrt {2\pi } {\sigma _v}}}\exp \left( { - {{\left( {{{\hat x}_v} - \left( {{x_f} + {\rm{\Delta }}{x_v}} \right)} \right)}^2}/2\sigma _v^2} \right) \times {1 \over {\sqrt {2\pi } {\sigma _p}}}\exp \left( { - {{\left( {{{\hat x}_p} - {x_f}} \right)}^2}/2\sigma _p^2} \right) \times {1 \over {\sqrt {2\pi } {\sigma _{pert}}}}\exp \left( { - {\rm{\Delta }}x_v^2/2\sigma _{pert}^2} \right) \times {1 \over {\sqrt {2\pi } {\sigma _f}}}\exp \left( { - {{\left( {{x_f} - \left( {{x_t} - b} \right)} \right)}^2}/2\sigma _f^2} \right)d{\rm{\Delta }}{x_v}\,d{x_f}.$$

The free parameters in this model are the bias and the *SD*s of the visual and proprioceptive noise.

#### Comparison observer

The next model compares the visual signal to the proprioceptive signal in order to make a decision. A perturbation is detected if the difference between the visual and proprioceptive estimates is sufficiently large, i.e., if

$$\newcommand{\bialpha}{\boldsymbol{\alpha}}\newcommand{\bibeta}{\boldsymbol{\beta}}\newcommand{\bigamma}{\boldsymbol{\gamma}}\newcommand{\bidelta}{\boldsymbol{\delta}}\newcommand{\bivarepsilon}{\boldsymbol{\varepsilon}}\newcommand{\bizeta}{\boldsymbol{\zeta}}\newcommand{\bieta}{\boldsymbol{\eta}}\newcommand{\bitheta}{\boldsymbol{\theta}}\newcommand{\biiota}{\boldsymbol{\iota}}\newcommand{\bikappa}{\boldsymbol{\kappa}}\newcommand{\bilambda}{\boldsymbol{\lambda}}\newcommand{\bimu}{\boldsymbol{\mu}}\newcommand{\binu}{\boldsymbol{\nu}}\newcommand{\bixi}{\boldsymbol{\xi}}\newcommand{\biomicron}{\boldsymbol{\micron}}\newcommand{\bipi}{\boldsymbol{\pi}}\newcommand{\birho}{\boldsymbol{\rho}}\newcommand{\bisigma}{\boldsymbol{\sigma}}\newcommand{\bitau}{\boldsymbol{\tau}}\newcommand{\biupsilon}{\boldsymbol{\upsilon}}\newcommand{\biphi}{\boldsymbol{\phi}}\newcommand{\bichi}{\boldsymbol{\chi}}\newcommand{\bipsi}{\boldsymbol{\psi}}\newcommand{\biomega}{\boldsymbol{\omega}}\left| {{{\hat x}_v} - {{\hat x}_p}} \right| = \left| {{\rm{\Delta }}{x_v} + {\varepsilon _v} - {\varepsilon _p}} \right| \gt C{\rm {.}}$$

The left hand side of this inequality has combined proprioceptive and visual variance ($\newcommand{\bialpha}{\boldsymbol{\alpha}}\newcommand{\bibeta}{\boldsymbol{\beta}}\newcommand{\bigamma}{\boldsymbol{\gamma}}\newcommand{\bidelta}{\boldsymbol{\delta}}\newcommand{\bivarepsilon}{\boldsymbol{\varepsilon}}\newcommand{\bizeta}{\boldsymbol{\zeta}}\newcommand{\bieta}{\boldsymbol{\eta}}\newcommand{\bitheta}{\boldsymbol{\theta}}\newcommand{\biiota}{\boldsymbol{\iota}}\newcommand{\bikappa}{\boldsymbol{\kappa}}\newcommand{\bilambda}{\boldsymbol{\lambda}}\newcommand{\bimu}{\boldsymbol{\mu}}\newcommand{\binu}{\boldsymbol{\nu}}\newcommand{\bixi}{\boldsymbol{\xi}}\newcommand{\biomicron}{\boldsymbol{\micron}}\newcommand{\bipi}{\boldsymbol{\pi}}\newcommand{\birho}{\boldsymbol{\rho}}\newcommand{\bisigma}{\boldsymbol{\sigma}}\newcommand{\bitau}{\boldsymbol{\tau}}\newcommand{\biupsilon}{\boldsymbol{\upsilon}}\newcommand{\biphi}{\boldsymbol{\phi}}\newcommand{\bichi}{\boldsymbol{\chi}}\newcommand{\bipsi}{\boldsymbol{\psi}}\newcommand{\biomega}{\boldsymbol{\omega}}\sigma _v^2 + \sigma _p^2$), which simplifies to the single free parameter $\newcommand{\bialpha}{\boldsymbol{\alpha}}\newcommand{\bibeta}{\boldsymbol{\beta}}\newcommand{\bigamma}{\boldsymbol{\gamma}}\newcommand{\bidelta}{\boldsymbol{\delta}}\newcommand{\bivarepsilon}{\boldsymbol{\varepsilon}}\newcommand{\bizeta}{\boldsymbol{\zeta}}\newcommand{\bieta}{\boldsymbol{\eta}}\newcommand{\bitheta}{\boldsymbol{\theta}}\newcommand{\biiota}{\boldsymbol{\iota}}\newcommand{\bikappa}{\boldsymbol{\kappa}}\newcommand{\bilambda}{\boldsymbol{\lambda}}\newcommand{\bimu}{\boldsymbol{\mu}}\newcommand{\binu}{\boldsymbol{\nu}}\newcommand{\bixi}{\boldsymbol{\xi}}\newcommand{\biomicron}{\boldsymbol{\micron}}\newcommand{\bipi}{\boldsymbol{\pi}}\newcommand{\birho}{\boldsymbol{\rho}}\newcommand{\bisigma}{\boldsymbol{\sigma}}\newcommand{\bitau}{\boldsymbol{\tau}}\newcommand{\biupsilon}{\boldsymbol{\upsilon}}\newcommand{\biphi}{\boldsymbol{\phi}}\newcommand{\bichi}{\boldsymbol{\chi}}\newcommand{\bipsi}{\boldsymbol{\psi}}\newcommand{\biomega}{\boldsymbol{\omega}}\sigma _{combined}^2$ because their individual contributions are not discriminable. The second and final free parameter $\newcommand{\bialpha}{\boldsymbol{\alpha}}\newcommand{\bibeta}{\boldsymbol{\beta}}\newcommand{\bigamma}{\boldsymbol{\gamma}}\newcommand{\bidelta}{\boldsymbol{\delta}}\newcommand{\bivarepsilon}{\boldsymbol{\varepsilon}}\newcommand{\bizeta}{\boldsymbol{\zeta}}\newcommand{\bieta}{\boldsymbol{\eta}}\newcommand{\bitheta}{\boldsymbol{\theta}}\newcommand{\biiota}{\boldsymbol{\iota}}\newcommand{\bikappa}{\boldsymbol{\kappa}}\newcommand{\bilambda}{\boldsymbol{\lambda}}\newcommand{\bimu}{\boldsymbol{\mu}}\newcommand{\binu}{\boldsymbol{\nu}}\newcommand{\bixi}{\boldsymbol{\xi}}\newcommand{\biomicron}{\boldsymbol{\micron}}\newcommand{\bipi}{\boldsymbol{\pi}}\newcommand{\birho}{\boldsymbol{\rho}}\newcommand{\bisigma}{\boldsymbol{\sigma}}\newcommand{\bitau}{\boldsymbol{\tau}}\newcommand{\biupsilon}{\boldsymbol{\upsilon}}\newcommand{\biphi}{\boldsymbol{\phi}}\newcommand{\bichi}{\boldsymbol{\chi}}\newcommand{\bipsi}{\boldsymbol{\psi}}\newcommand{\biomega}{\boldsymbol{\omega}}C$ is the threshold perturbation magnitude (in units of motor error *SD*) for which the probability of the detection response is 50%. There is no bias parameter in this model because bias does not enter into the comparison between the proprioceptive and visual signals.

#### Visual-cue-only observer

Our final observer's responses are based on visually displayed feedback alone, and disregard estimates of reach endpoint from proprioception. This observer detects a perturbation when visual feedback of fingertip position differs sufficiently from the expected endpoint, or

$$\newcommand{\bialpha}{\boldsymbol{\alpha}}\newcommand{\bibeta}{\boldsymbol{\beta}}\newcommand{\bigamma}{\boldsymbol{\gamma}}\newcommand{\bidelta}{\boldsymbol{\delta}}\newcommand{\bivarepsilon}{\boldsymbol{\varepsilon}}\newcommand{\bizeta}{\boldsymbol{\zeta}}\newcommand{\bieta}{\boldsymbol{\eta}}\newcommand{\bitheta}{\boldsymbol{\theta}}\newcommand{\biiota}{\boldsymbol{\iota}}\newcommand{\bikappa}{\boldsymbol{\kappa}}\newcommand{\bilambda}{\boldsymbol{\lambda}}\newcommand{\bimu}{\boldsymbol{\mu}}\newcommand{\binu}{\boldsymbol{\nu}}\newcommand{\bixi}{\boldsymbol{\xi}}\newcommand{\biomicron}{\boldsymbol{\micron}}\newcommand{\bipi}{\boldsymbol{\pi}}\newcommand{\birho}{\boldsymbol{\rho}}\newcommand{\bisigma}{\boldsymbol{\sigma}}\newcommand{\bitau}{\boldsymbol{\tau}}\newcommand{\biupsilon}{\boldsymbol{\upsilon}}\newcommand{\biphi}{\boldsymbol{\phi}}\newcommand{\bichi}{\boldsymbol{\chi}}\newcommand{\bipsi}{\boldsymbol{\psi}}\newcommand{\biomega}{\boldsymbol{\omega}}\left| {{{\hat x}_v} - \left( {{{\hat x}_t} - b} \right)} \right| \gt C{\rm {.}}$$

This model is similar to the *comparison observer*, again with a combined noise parameter (in this case effectively doubling the visual noise), except that the bias parameter $\newcommand{\bialpha}{\boldsymbol{\alpha}}\newcommand{\bibeta}{\boldsymbol{\beta}}\newcommand{\bigamma}{\boldsymbol{\gamma}}\newcommand{\bidelta}{\boldsymbol{\delta}}\newcommand{\bivarepsilon}{\boldsymbol{\varepsilon}}\newcommand{\bizeta}{\boldsymbol{\zeta}}\newcommand{\bieta}{\boldsymbol{\eta}}\newcommand{\bitheta}{\boldsymbol{\theta}}\newcommand{\biiota}{\boldsymbol{\iota}}\newcommand{\bikappa}{\boldsymbol{\kappa}}\newcommand{\bilambda}{\boldsymbol{\lambda}}\newcommand{\bimu}{\boldsymbol{\mu}}\newcommand{\binu}{\boldsymbol{\nu}}\newcommand{\bixi}{\boldsymbol{\xi}}\newcommand{\biomicron}{\boldsymbol{\micron}}\newcommand{\bipi}{\boldsymbol{\pi}}\newcommand{\birho}{\boldsymbol{\rho}}\newcommand{\bisigma}{\boldsymbol{\sigma}}\newcommand{\bitau}{\boldsymbol{\tau}}\newcommand{\biupsilon}{\boldsymbol{\upsilon}}\newcommand{\biphi}{\boldsymbol{\phi}}\newcommand{\bichi}{\boldsymbol{\chi}}\newcommand{\bipsi}{\boldsymbol{\psi}}\newcommand{\biomega}{\boldsymbol{\omega}}b$ appears here. It has three free parameters.

We fit the models by evaluating the likelihood at each point in a parameter grid. For each condition (gain and direction perturbation), we normalized the likelihood and multiplied by a weak (standard normal) prior on the parameters to get the resulting posterior. We randomly sampled from this posterior 1000 times, and used the 2.5th and 97.5th percentiles as confidence bounds on the fit parameters. To compare models quantitatively, we calculated the Akaike Information Criterion (Akaike, 1974) using the parameter values that resulted in maximum posterior probability (MAP). The priors did not largely affect the best-fitting parameters, nor did they change the relative AIC values.

The magnitude of subjects' variable motor error decreased over sessions, indicating learning. Therefore, reach endpoint errors and perturbation magnitudes used in this analysis were first normalized by the standard deviation of reach endpoint error from the sessions in which they were collected. This method also has the benefit of normalizing across subjects and describes the perturbations and the errors in both the gain- and the direction-perturbation sessions using the same units. All analyses were carried out in these session-specific *SD* units.

## Results

Subjects made center-out reaches to a visual target. Reach endpoint feedback was provided. On half of the trials feedback was perturbed either in gain or direction. We sought to determine how people use visual and proprioceptive cues to detect the perturbation.

**Figure 2:**
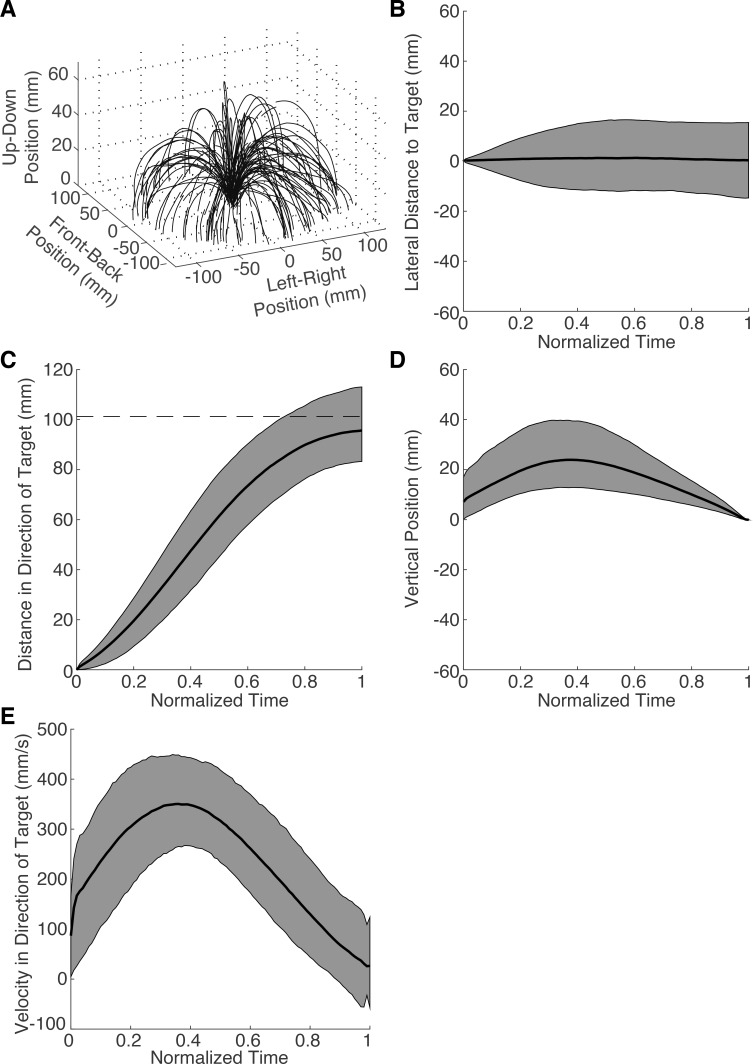
Raw data of Subject 3's gain-perturbation sessions. (A) Trajectories of the first 100 trials. The vertical axis scale is exaggerated for clarity. Position in the direction lateral to the target (B), in the direction of the target (C), and vertically (D), and velocity in the direction of the target (E) are plotted as a function of normalized time. Black curve: median for all 2000 trajectories. Dashed line in C: target distance. Gray: 95% CI.

Reach trajectory kinematics were inspected to ensure that there were no obvious defects in the data collection process. Figure 2A shows the first 100 trials from Subject 3 during the first gain-perturbation session. The trajectories have an arching shape, as instructed, and the invisible circle on which the targets appeared is discernible. Figures 2B through D show the median and 95% confidence regions of trajectory positions in each of the three dimensions relative to the target location. Trajectories were normalized into 100 time bins using a linear interpolation of the raw positions. Trajectories followed a straight path to the target (Figure 2B) with the typical reach profile, arching up off the table and then back down (Figure 2C through D). They also followed a typical velocity profile (Figure 2E).

**Figure 3:**
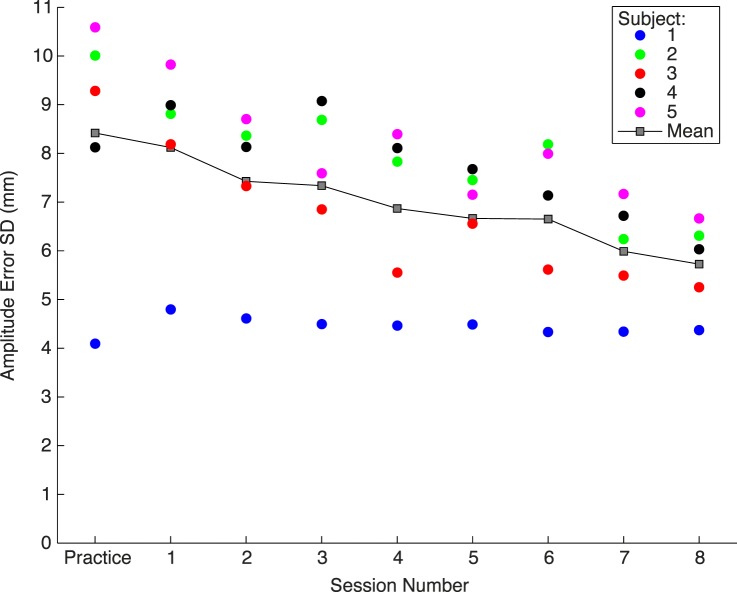
Reach precision (SD of movement amplitude in mm) as a function of session number. Precision improved across sessions. Data are from the gain-perturbation sessions. Subject 1 (author EGC) was experienced with the setup. Gray squares: mean across subjects.

We first asked if subjects exhibited motor learning by analyzing trajectory endpoint errors over the course of the experiment. Specifically, the *SD* of finger endpoint errors continued to decrease after the initial learning session (Figure 3). A Pearson correlation between session number (1–8) and *SD* of gain error, averaged across subjects (gray boxes in Figure 3), confirms the presence of motor learning (*r* = −0.97, *p* < 0.0001). This was initial justification for normalizing the data within each session, specifying all locations in units of that session's motor *SD* (see Methods).

**Figure 4:**
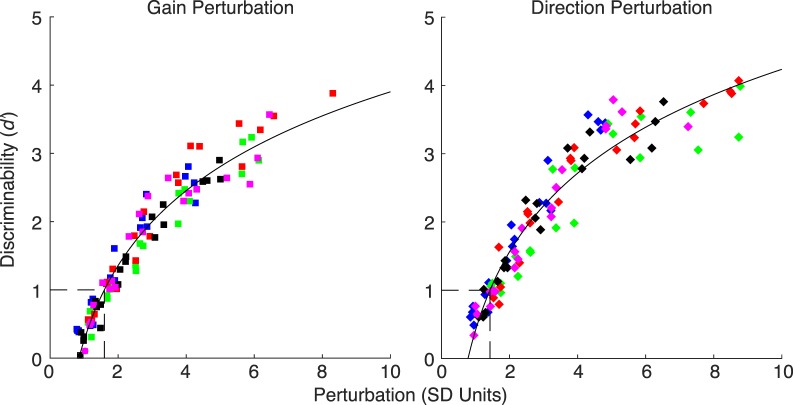
Performance (d′) as a function of perturbation magnitude for the gain- and direction-perturbation sessions. Data are plotted separately for each subject, session and perturbation level. Perturbation values are plotted in SD units of endpoint error from each session separately. The colors correspond to subjects, as in Figure 3. Solid curve: logarithmic fit.

Using these session-specific perturbation values, we asked how subjects' performance varied as a function of the perturbation. As expected, the ability to detect the perturbation increased monotonically with the strength of the perturbation (Figure 4). We calculated separate *d*′ values for each perturbation level using the hit rate for that perturbation level and the common no-perturbation false alarm rate. An additional correction was made to the *d*′ value for hit and false alarm rates of 1 or 0 (Hautus, 1995). Figure 4 includes 20 perturbation values (5 absolute perturbation levels by 4 sessions) for each subject and axis of perturbation, so that each hit rate is based on 50 trials.

We next determined the magnitude of perturbation that subjects can reliably detect. We wished to interpolate the data to find the perturbation corresponding to *d*′ = 1. By eye, we found that a logarithmic curve of the form $\newcommand{\bialpha}{\boldsymbol{\alpha}}\newcommand{\bibeta}{\boldsymbol{\beta}}\newcommand{\bigamma}{\boldsymbol{\gamma}}\newcommand{\bidelta}{\boldsymbol{\delta}}\newcommand{\bivarepsilon}{\boldsymbol{\varepsilon}}\newcommand{\bizeta}{\boldsymbol{\zeta}}\newcommand{\bieta}{\boldsymbol{\eta}}\newcommand{\bitheta}{\boldsymbol{\theta}}\newcommand{\biiota}{\boldsymbol{\iota}}\newcommand{\bikappa}{\boldsymbol{\kappa}}\newcommand{\bilambda}{\boldsymbol{\lambda}}\newcommand{\bimu}{\boldsymbol{\mu}}\newcommand{\binu}{\boldsymbol{\nu}}\newcommand{\bixi}{\boldsymbol{\xi}}\newcommand{\biomicron}{\boldsymbol{\micron}}\newcommand{\bipi}{\boldsymbol{\pi}}\newcommand{\birho}{\boldsymbol{\rho}}\newcommand{\bisigma}{\boldsymbol{\sigma}}\newcommand{\bitau}{\boldsymbol{\tau}}\newcommand{\biupsilon}{\boldsymbol{\upsilon}}\newcommand{\biphi}{\boldsymbol{\phi}}\newcommand{\bichi}{\boldsymbol{\chi}}\newcommand{\bipsi}{\boldsymbol{\psi}}\newcommand{\biomega}{\boldsymbol{\omega}}y = a\log x + b$ fit the data well, although this function has no particular theoretical significance. Figure 4 shows the fits (ordinary least squares) separately for the gain- and direction-perturbation sessions. Thresholds were 1.59 and 1.43 *SD* units in the gain and direction conditions, respectively. Individual subject thresholds are shown in Table A1. Thus, a perturbation is reliably detected (approximately 69% correct responses if subjects are maximizing percent correct) when its magnitude is about 1.5 times a typical reach error. We found further justification for normalizing data into *SD* units by fitting a function to the raw data in millimeters. Fits to the normalized data were better. For gain and dimension perturbations, *r*^2^ = 0.93 and *r*^2^ = 0.92 for the normalized data, and *r*^2^ = 0.76 and *r*^2^ = 0.80 for the unnormalized data. Therefore, all subsequent analyses are in *SD* units.

**Figure 5:**
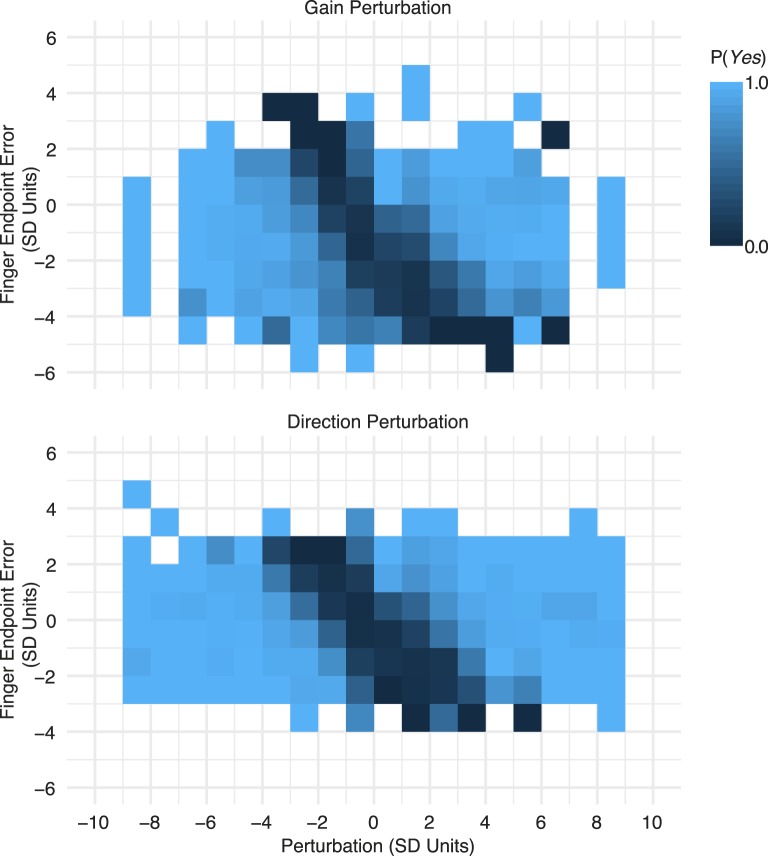
Response data. The color indicates the proportion of trials in which subjects responded Yes, binned by perturbation value and reach endpoint error. Data from all subjects are pooled. Axes are in units of reach endpoint SD.

**Figure 6:**
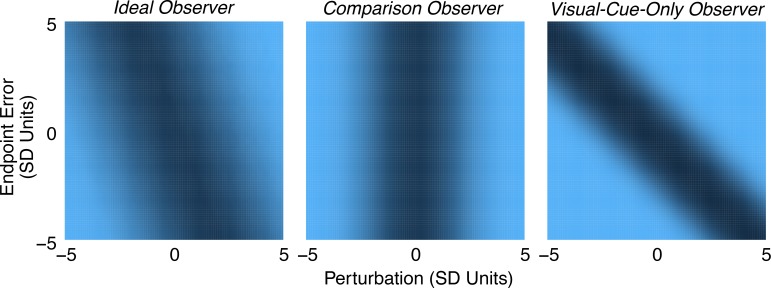
Observer model fits. Probability of a Yes response as a function of perturbation and reach endpoint error as predicted by each of three models. The parameter values used for each model are from maximum a posteriori fits to the data of Subject 3 in the gain-perturbation session, indicated in Figure A1 with an asterisk. The color scale matches that of Figure 5.

**Figure A1:**
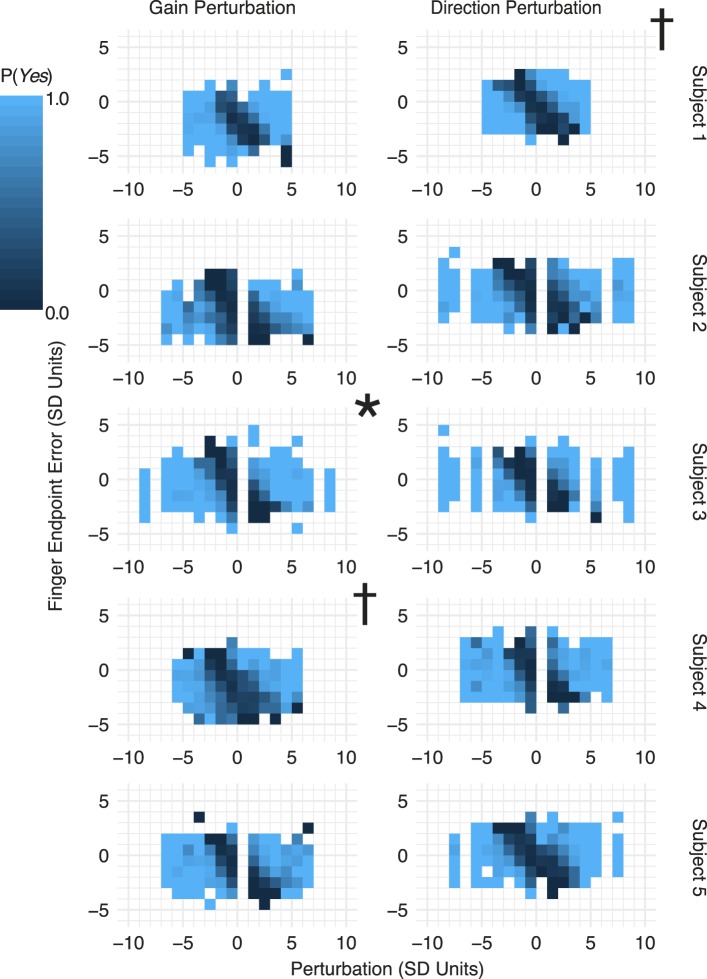
Individual response data. These are the same data as in Figure 5, but split by subject. Some subjects, such as subject 1, exhibited systematic hypometria, as shown by the shift of data towards negative endpoint errors during the gain perturbation sessions (top left panel). The asterisk indicates the data whose fitted models are displayed in Figure 6. The daggers indicate the two cases where the data were best fit by the ideal observer.

Next, we analyzed how sensory cues are combined to make the perturbation-detection decision. Figure 5 shows heatmaps of the proportion of *Yes* responses for each combination of perturbation and reach endpoint error (binned). The axes are in units of *SD* of the subject's own motor noise, as in Figure 4. The raw data, which are shown binned in Figure 5, were used to determine which model best captured subjects' strategies. Figure 6 shows each model with the best-fitting parameters for one example subject and condition (data indicated by an asterisk in Figure A1). Intuitively, an observer should compare the sensed finger endpoint position (based on proprioceptive signals) with visual feedback of the finger position, and respond *Yes* when the difference between these position measurements is sufficiently large. In the coordinates of Figures 5 and 6, this strategy predicts a lower proportion of *Yes* responses when the feedback was not perturbed (i.e., a trough aligned with Perturbation = 0). The sub-optimal *comparison observer* (middle panel in Figure 6) depicts this prediction. The *visual*-*cue*-*only observer*, on the other hand, ignores the proprioceptive signal and responds *Yes* based solely on the distance of the feedback from the target (figuring in expected pointing bias). The corresponding trough (right panel in Figure 6) is aligned with the diagonal axis, resulting in a minimum probability of saying *Yes* when the perturbation is equal and opposite to the endpoint error, putting the feedback at the location of the target. The *ideal observer* (left panel in Figure 6) combines the sources of sensory noise in a statistically optimal way. The orientation of the trough falls somewhere in between the other two models.

**Figure 7:**
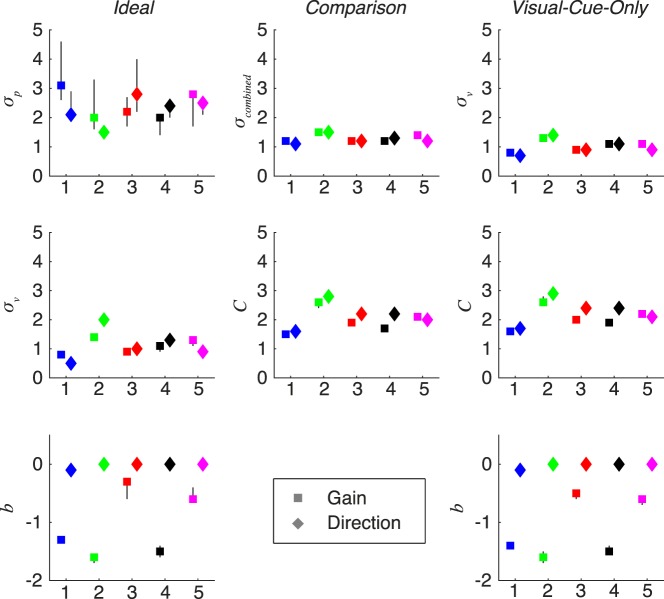
MAP estimates of the parameters for each subject (indicated by x axis index), perturbation dimension, and model. Error bars: 95% CI (samples from the posterior), some of which are occluded by the MAP marker. Colors correspond to subject, as in Figure 3.

The MAP estimates of the parameters and their 95% CI for each model, subject, and perturbation session are shown in Figure 7 (see Methods: Models). The parameter $\newcommand{\bialpha}{\boldsymbol{\alpha}}\newcommand{\bibeta}{\boldsymbol{\beta}}\newcommand{\bigamma}{\boldsymbol{\gamma}}\newcommand{\bidelta}{\boldsymbol{\delta}}\newcommand{\bivarepsilon}{\boldsymbol{\varepsilon}}\newcommand{\bizeta}{\boldsymbol{\zeta}}\newcommand{\bieta}{\boldsymbol{\eta}}\newcommand{\bitheta}{\boldsymbol{\theta}}\newcommand{\biiota}{\boldsymbol{\iota}}\newcommand{\bikappa}{\boldsymbol{\kappa}}\newcommand{\bilambda}{\boldsymbol{\lambda}}\newcommand{\bimu}{\boldsymbol{\mu}}\newcommand{\binu}{\boldsymbol{\nu}}\newcommand{\bixi}{\boldsymbol{\xi}}\newcommand{\biomicron}{\boldsymbol{\micron}}\newcommand{\bipi}{\boldsymbol{\pi}}\newcommand{\birho}{\boldsymbol{\rho}}\newcommand{\bisigma}{\boldsymbol{\sigma}}\newcommand{\bitau}{\boldsymbol{\tau}}\newcommand{\biupsilon}{\boldsymbol{\upsilon}}\newcommand{\biphi}{\boldsymbol{\phi}}\newcommand{\bichi}{\boldsymbol{\chi}}\newcommand{\bipsi}{\boldsymbol{\psi}}\newcommand{\biomega}{\boldsymbol{\omega}}C$, used by the *comparison* and *visual*-*cue*-*only observers*, represents the number of *SD*s of motor noise in the difference between the comparison signals (i.e., between the visual feedback and the proprioceptively felt hand position for the former, and the visual feedback and the target position for the latter) before subjects begin responding *Yes*. $\newcommand{\bialpha}{\boldsymbol{\alpha}}\newcommand{\bibeta}{\boldsymbol{\beta}}\newcommand{\bigamma}{\boldsymbol{\gamma}}\newcommand{\bidelta}{\boldsymbol{\delta}}\newcommand{\bivarepsilon}{\boldsymbol{\varepsilon}}\newcommand{\bizeta}{\boldsymbol{\zeta}}\newcommand{\bieta}{\boldsymbol{\eta}}\newcommand{\bitheta}{\boldsymbol{\theta}}\newcommand{\biiota}{\boldsymbol{\iota}}\newcommand{\bikappa}{\boldsymbol{\kappa}}\newcommand{\bilambda}{\boldsymbol{\lambda}}\newcommand{\bimu}{\boldsymbol{\mu}}\newcommand{\binu}{\boldsymbol{\nu}}\newcommand{\bixi}{\boldsymbol{\xi}}\newcommand{\biomicron}{\boldsymbol{\micron}}\newcommand{\bipi}{\boldsymbol{\pi}}\newcommand{\birho}{\boldsymbol{\rho}}\newcommand{\bisigma}{\boldsymbol{\sigma}}\newcommand{\bitau}{\boldsymbol{\tau}}\newcommand{\biupsilon}{\boldsymbol{\upsilon}}\newcommand{\biphi}{\boldsymbol{\phi}}\newcommand{\bichi}{\boldsymbol{\chi}}\newcommand{\bipsi}{\boldsymbol{\psi}}\newcommand{\biomega}{\boldsymbol{\omega}}C$ is the difference in the comparison signals at which subjects respond *Yes* half the time. Because of the reasonable range of parameter values (approximately 1.5–3 *SD*s), the remarkable consistency within subject and between models and their tiny confidence intervals, we can be confident in these estimates.

**Figure 8:**
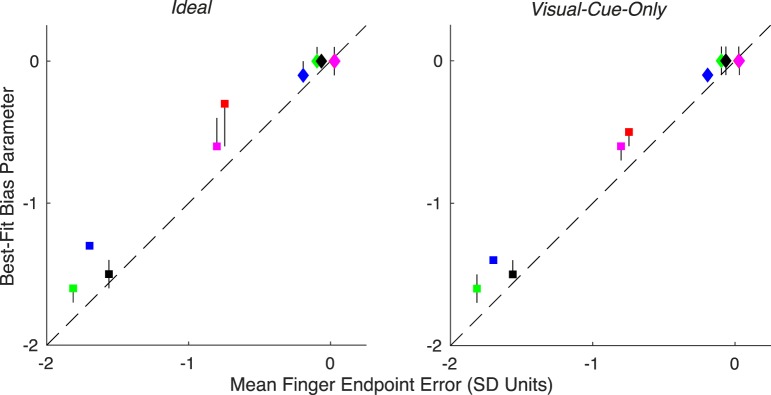
Correlation between endpoint error and bias parameter. This parameter estimates the endpoint error during no-perturbation trials for which the subject is least likely to respond Yes. Endpoint errors and bias values are plotted in session-specific SD units. Colors and shapes correspond to subject and session, as in Figure 7. Vertical error bars represent 95% CI of the sampled posteriors, and some are occluded by the markers. Horizontal error bars are present and represent the SEM, but all are occluded by the markers. In some cases, vertical errors bars overlap with the identity line, indicating that those subjects had and used an accurate internal model of mean endpoint error. In the rest of the cases, the bias parameter was less than the endpoint error, indicating that those subjects did not fully incorporate mean endpoint error into responses.

Another interesting feature of the parameters is the pattern in the observers' bias. The bias accounts for subjects' recognition of the average motor error when making the detection decision, and therefore this parameter is a stand-in for subjects' internal model of motor error. Across all models and subjects, the sessions where the perturbation was applied to reach direction led to a best-fit bias parameter that was zero or close to zero. This makes sense because reach endpoint errors were not biased clockwise or counterclockwise of the target. However, all subjects exhibited at least some degree of hypometria, meaning that they undershot the target. The first evidence in favor of subjects having an accurate internal model of endpoint error distribution is the fact that the best-fitting bias parameters were always negative when the reach feedback was perturbed in gain. They are negative because subjects undershot the target, and are only negative during the gain sessions because undershooting the target is irrelevant for detecting a perturbation of reach direction. The best-fitting bias parameters for the gain-perturbation sessions for the two models that have this parameter are similar for each subject (Figure 7). The bias parameter will shift each observer's trough along the Perturbation = 0 axis, which does not change the shape of the heatmap. More evidence for subjects' accurate internal model of endpoint error distribution comes from a quantitative comparison between subjects' best-fit bias parameter and their actual mean endpoint error (Figure 8). An accurate internal model predicts that the bias parameter equals the mean endpoint error (i.e., the data fall on the identity line). For both the *ideal* and the *visual*-*cue*-*only observers*, there is a high correlation in the gain-perturbation sessions *r* = 0.966, *p* < 0.01 and *r* = 0.986, *p* < 0.01, respectively). These did not reach significance in the direction-perturbation sessions (*r* = 0.803, *p* = 0.1 and *r* = 0.803, *p* = 0.1), for which there was little variance in either the bias parameters or the mean endpoint errors across subjects. Additionally, it is primarily the fits from the direction-perturbation sessions that have confidence intervals that overlap with the identity line. For the majority of cases (9/10), the bias parameters from the gain-perturbation sessions were slightly less than the actual mean endpoint error. This indicates that subjects either believed their mean endpoint to be slightly closer to the target than it actually was, or that they did not fully incorporate knowledge of their mean endpoint into their detection decisions.

**Figure 9:**
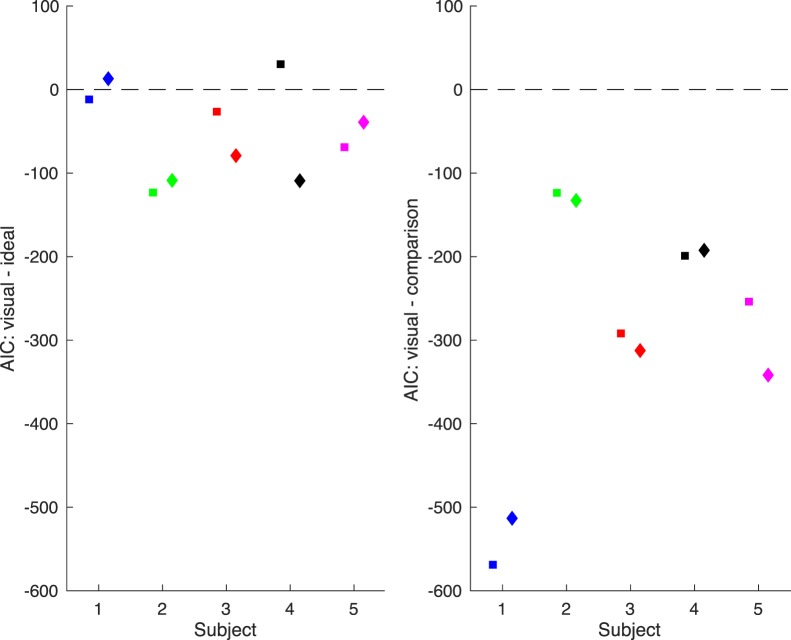
Model comparison. Differences between AIC values for the visual-cue-only and ideal observers, and for the visual-cue-only and comparison observers. Negative values indicate that the visual-cue-only observer fit the data better, even when including a penalty for having more free parameters in the case of the ideal observer. By and large, the visual-cue-only observer dominated the other models. Only in two cases was it not the best fitting model and in those cases, the subject was best fit by the ideal observer for only one of the perturbation dimension sessions.

The *visual*-*cue*-*only observer* was more representative of subjects' behavior than either of the other models. AIC values were consistently lower for that model than for the others (Figure 9), indicating a better fit, despite the penalty for having more free parameters. For two of the subjects, data from just one of the dimensions of perturbation were better fit by the *ideal observer*. This means that to a great degree, subjects responded *No* when the perturbation was equal and opposite the reach error (i.e., when the displayed feedback was on target). We therefore conclude that subjects ignored proprioception and respond based solely on the final visual feedback.

## Discussion

In this experiment, endpoint feedback of center-out reaches was perturbed to determine how subjects combined proprioceptive and endpoint feedback signals to detect perturbation. We do not have access to subjects' proprioceptive estimates of reach endpoints. Rather, we used perturbation magnitude and endpoint error to predict subjects' responses. We found that subjects are reasonably successful at detecting perturbations (Figure 4), but primarily rely on the displayed endpoint error (Figure 9). An *ideal observer* optimally combines noisy proprioceptive and visual signals and responds primarily on perturbation magnitude, not on the displayed magnitude of endpoint error (left panel in Figure 6). The experimental data more closely resembled a *visual*-*cue*-*only observer,* who responds based only on the feedback location relative to the target. This feedback is a combination of the endpoint error and the perturbation, and ignores information regarding the proprioceptive signal. Our findings corroborate those of Kluzik et al. (2008), who showed that smaller errors will update an internal model of the arm rather than of an external tool, just as our data show that subjects attributed small visual errors to themselves.

We found that the threshold to detect a perturbation (i.e., *d*′ = 1 and approximately 69% correct if maximizing percent correct) was about 1.5 times the subject's intrinsic motor *SD*. This value is independent of any *Yes*/*No* bias, and is therefore the best subjects could perform given their suboptimal strategy. The relationship between perturbation size and detectability of the perturbation was highly consistent across sessions (Figure 4 and Table A1). The fact that this correlation was greater for perturbations in *SD*s of motor noise than for perturbations measured in millimeters suggests that subjects (knowingly or unknowingly) incorporated their typical motor error from each session into their decisions, as measured by *d*′.

While the findings in the current study can inform future motor-adaptation experiments, we deliberately minimized any effects of reach adaptation by applying perturbations that were random in sign and magnitude. It might not be possible to prevent subjects from adapting to perturbed feedback considering the evidence for trial-by-trial correction (Baddeley, Ingram, & Miall, 2003; Scheidt, Dingwell, & Mussa-Ivaldi, 2001; van Beers, 2009; Wei & Körding, 2009). It is unclear how an *ideal observer* would behave in this task if adapted to a perturbation, but adaptation should not play a role for the *visual*-*cue*-*only observer* because it disregards the proprioceptive signal.

A parsimonious explanation for our findings is that it was easier for subjects to ignore the proprioceptive cue. The concept of visual capture is consistent with the overwhelming contribution of vision to subjects' decisions (Rock & Victor, 1964). Furthermore, we may have biased subjects to prioritize vision by providing veridical visual feedback (Figure 1B) and not proprioceptive feedback (for example, by having subjects touch the correct location of the target after the trial). Indeed, there are studies of deafferented individuals that show proprioception is not required to allow someone to adapt to a visually observed motor perturbation (Bard, Fleury, Teasdale, Paillard, & Nougier, 1995; Ingram et al., 2000; Yousif, Cole, Rothwell, & Diedrichsen, 2015). However, this is contrary to the typical finding that subjects try to optimize performance, which requires subjects to use the proprioceptive signal. More concretely, there is plenty of evidence that subjects optimally combine proprioceptive and visual signals in motor tasks (e.g., Niemeier et al., 2003; Sober & Sabes, 2003, 2005; van Beers, Sittig, et al., 1999; van Beers, Wolpert, et al., 2002; van Dam & Ernst, 2013). Most of the evidence for optimal integration comes from an analysis of motor output alone. An exception to this is van Dam and Ernst (2013), wherein subjects made secondary, corrective movements to the target, as well as subsequent verbal reports about the direction of the residual error after the corrective movement. They provided evidence that subjects optimally combined the proprioceptive signal and motor variability in making the corrective movement. However, they were no better than chance at discriminating the direction of the subsequent residual error. Our findings stand in direct contrast to theirs.

The conflict between findings of optimal integration and the current results hints at the dissociation between perception for action and perception for recognition (e.g., Aglioti, DeSouza, & Goodale, 1995; Goodale, Pélisson, & Prablanc, 1986). Our results are evidence that sensory signals (or transformed signals) are not always accessible to explicit reports. On the other hand, when sensory signals are used for motor planning, they are optimally integrated, as in the corrective reaches in van Dam and Ernst (2013). The fact that van Dam and Ernst also found evidence for optimal behavior using explicit reports, while we did not, indicates that optimality can be achieved easily in some tasks but not others, even if those tasks and cost functions are seemingly similar (e.g., Wu, Trommershäuser, Maloney, & Landy, 2006).

To detect a perturbation using the proprioceptive signal, subjects in our task had to compare visual feedback with the hand position determined by an internal model. This necessitates a coordinate transformation between display and tabletop (Figure 1A). It is possible that carrying out this transformation involves an additional source of noise that interferes with the ability to compare the two signals (van der Graaff, Brenner, & Smeets, 2016). However, Parmar, Huang, and Patton (2011) found this not to be the case, and van Dam and Ernst (2013) found that this transformation merely increased proprioceptive noise but did not change subjects' behavior. As in van Dam and Ernst, the transformation merely adds to the proprioceptive noise. In our study, additional proprioceptive noise would not cause *ideal observer* performance to resemble that of the *visual*-*cue*-*only observer*. Therefore, we can be confident that our findings are unchanged by the tabletop-to-monitor transformation.

Some studies explicitly claim that subjects are unaware of much larger perturbations (e.g., Sawers et al., 2013; Werner et al., 2015), and it is also common for there to be no mention of a subject's awareness of such perturbations (e.g., Ghez, Scheidt, & Heijink, 2007). A key difference with our study is that the nature of our task primed subjects to be cognizant of the potential presence of a perturbation. While many studies have used small or gradually increasing perturbations to avoid subjects' awareness, Kagerer and colleagues (1997) describe an extreme situation, where a directional perturbation increased in 10° steps up to 90°. For comparison, during the direction session of the current study, subjects could reliably detect perturbations between 3.6° and 6.6°. Those authors report that the perturbation went undetected by the subjects, presumably because they were not primed to do so for the task. It seems highly unlikely that even naive subjects would be completely unaware of a 90° rotation of visual feedback. A more plausible description is that incrementally increasing perturbation increases the detection threshold by some amount. Furthermore, in naturalistic settings, people are only sometimes aware of both internal and external constraints placed on motor plans, such as due to fatigue or an inaccurate estimation of an object's weight. However, our findings are not necessarily in conflict with Kagerer et al. because of the difference in subjects' expectations; the differences in these studies emphasize the qualitative difference between cognitive awareness and detection threshold. Our findings, in conjunction with those that describe the differences in motor compensation to a noticed or unnoticed perturbation (Huberdeau et al., 2015; Hwang et al., 2006), highlight the importance of subjects' expectations and awareness of perturbation.

One-shot reaches with no online visual feedback, as performed in this study, are atypical in the natural world. No movement is performed in isolation, and knowledge of the end effector's starting position is typically crucial for planning a movement. In this study, the veridical initial position of the reaching finger was provided on the display. A possible experimental method for forcing incorporation of proprioceptive signals into decisions (both verbal and motor) would be to have subjects make a series of sequential movements without visual feedback. In this case, the motor plan for movement *n* + 1 is dependent on noisy proprioceptive information garnered during movement *n*.

The current study provides a tool for building an experimental task to maximize the signal-to-noise ratio when estimating adaptation despite trial-to-trial endpoint variability. This is important in experiments that require isolation of implicit (e.g., adaptive compensation) from explicit (e.g., detection; deliberate correction) motor processes. Tasks designed to elicit motor compensation can illuminate the goals and computations carried out by the sensorimotor system. The role and contribution of a subject's awareness of motor errors in both the formulation and correction of motor plans is just beginning to be investigated (Benson, Anguera, & Seidler, 2011; Hwang et al., 2006; Mazzoni & Krakauer, 2006; Niemeier et al., 2003; van Dam & Ernst, 2013), and the current study hints at a level of complexity in these relationships, in light of the suboptimalities in signal integration demonstrated here.

## Conclusions

We measured detection threshold of perturbed visual feedback of reaches. We found that people can detect perturbations about 1.5 times their trial-to-trial motor variability. A model comparison showed that people ignore proprioception when deciding whether or not they had been perturbed; they rely solely on the final visual feedback.

## Figures and Tables

**Figure A2:**
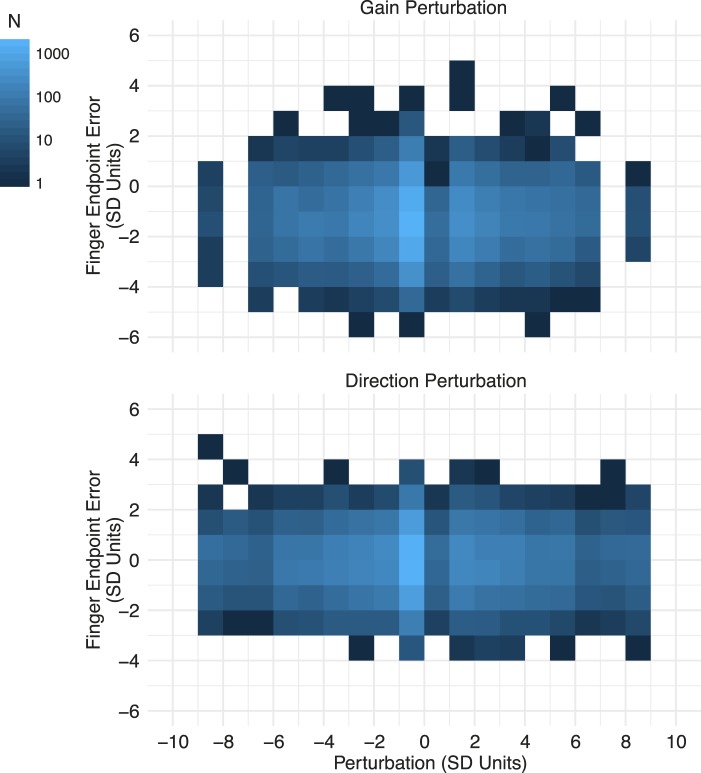
Trials per bin. Horizontal and vertical axes are the same as in Figure 5, but color here indicates the number of trials per bin.

## Appendix

**Table A1:** Perturbation-detection thresholds computed for subjects individually in the same manner as the group fit in Figure 4.

Subject	Gain	Direction
1	1.42	1.31
2	1.81	1.61
3	1.62	1.52
4	1.67	1.45
5	1.60	1.48
Mean (*SEM*)	1.62 (0.06)	1.47 (0.05)
